# Influence of wet distillers grains diets on beef cattle fecal bacterial community structure

**DOI:** 10.1186/1471-2180-12-25

**Published:** 2012-02-24

**Authors:** William C Rice, Michael L Galyean, Stephen B Cox, Scot E Dowd, N Andy Cole

**Affiliations:** 1 Conservation and Production Research Laboratory, USDA ARS, Bushland, TX 79012, USA; 2 Department of Animal and Food Sciences, Texas Tech University, Lubbock, TX 79409, USA; 3 Research and Testing Laboratories, 1004 Garfield Dr. Building #340, Lubbock, TX 79416, USA; 4 Molecular Research (MR DNA), 503 Clovis Road, Shallowater, TX 79363, USA

## Abstract

**Background:**

The high demand for ethanol in the U.S. has generated large stocks of wet distillers grains (DG), a byproduct from the manufacture of ethanol from corn and sorghum grains. Little is known, however, about the potential influence of dietary DG on fecal microbial community structure. A better understanding of the microbial population in beef cattle feces could be an important monitoring tool to facilitate goals of improving nutrient management, increasing animal growth performance and decreasing odors and/or shedding of pathogens. Five diets consisting of a traditional diet fed to finishing beef cattle in the Southern High Plains of Texas-CON (steam-flaked corn control with 0% DG), and four concentrations of DG in the dietary dry matter; 10 C (10% corn-based DG), 5S (5% sorghum-based DG), 10S (10% sorghum DG), and 15S (15% sorghum DG) were fed to steers at the Texas Tech University Burnett Animal Center. Diets were essentially isonitrogenous with a formulated crude protein value of 13.5%.

**Results:**

Fecal grab samples were obtained from 20 steers (n = 4 per diet) and the barcoded DNA pyrosequencing method was used to generate 127,530 16S operational taxonomic units (OTUs). A total of 24 phyla were observed, distributed amongst all beef cattle on all diets, revealing considerable animal to animal variation, however only six phyla (core set) were observed in all animals regardless of dietary treatment. The average abundance and range of abundance, respectively of the core phyla were as follows: Firmicutes (61%, 19 to 83%), Bacteroidetes (28%, 11 to 63%), Proteobacteria (3%, 0.34 to 17.5%), Tenericutes (0.15%, 0.0 to 0.35%), Nitrospirae (0.11%, 0.03 to 0.22%), and Fusobacteria (0.086%, 0.017 to 0.38%). Feeding DG-based diets resulted in significant shifts in the fecal microbial community structure compared with the traditional CON. Four low abundance phyla significantly responded to dietary treatments: Synergistetes (*p *= 0.01), WS3 (*p *= 0.054), Actinobacteria (*p *= 0.06), and Spirochaetes (*p *= 0.06).

**Conclusions:**

This is, to our knowledge, the first study using this method to survey the fecal microbiome of beef cattle fed various concentrations of wet DG. Comparison of our results with other cattle DNA sequencing studies of beef and dairy cattle feces from a variety of geographical locations and different management practices identifies a core set of three phyla shared across all cattle. These three phyla, in order of relative abundance are; Firmicutes, Bacteroidetes, and Proteobacteria. The presence of large animal-to-animal variation in cattle microbiome was noted in our study as well as by others.

## Background

The high demand for ethanol in the U.S. has generated large stocks of wet distillers grains (DG) derived as a byproduct from the manufacture of ethanol from corn and sorghum grains. Ethanol production is expected to increase several fold due to the high demand and cost of foreign oil [1]. Energy and protein dense DGs are attractive for use as a feed for beef cattle finishing diets; however little is known about the potential influence of dietary DG on fecal microbial community structure. A better understanding of the microbial population in beef cattle feces could be important in improving nutrient management, increasing animal growth performance, and decreasing odors and/or shedding of pathogens. A variety of emissions such as ammonia, volatile fatty acids, and hundreds of volatile organic compounds [2] have been tied to beef cattle manure (reviewed by [3-5]). Volatilization of ammonia has been linked to crude protein content in the diet fed and increased amounts of excreted urinary N [6]. Previous studies suggested an association between dried distillers grains (DDGS) feeding and an increased prevalence and fecal shedding of the foodborne pathogen *Escherichia coli *O157:H7 in cattle [7-9].

A small number of studies have used culture-independent 16S rRNA-based [10] and culture-dependent 16S rRNA-based methods with dairy cattle feces [11,12]. *Clostridium *spp were identified as the most dominant taxa across all lactating dairy cows (19% average abundance, range 13.9-25.4%) followed by *Bacteroides *spp (9.26%, 5.2-13.7% respectively) using the culture-independent approach [10]. In this study of Holstein dairy cows (n = 20), 274 different bacterial species were detected corresponding to 142 separate genera [10]. Several thousand sequences were obtained per sample enabling the detection of populations below 0.1% abundance. Using culture-dependent methods, a total of 284 16S rRNA clones were obtained from three Holstein steers and classified at the 98% sequence similarity level [12]. The dominant phyla observed were: Firmicutes (81.3%), Bacteroidetes (14.4%), Actinobacteria (2.5%), and Proteobacteria (1.4%). A comparison of dairy cattle fed a control diet or fed a diet supplemented with monensin using the culture-dependent 16S rRNA method returned 6,912 16S rRNA genes [11]. Nearly equivalent abundance levels of Firmicutes (36.4-46.5%) and Bacteroidetes (40.5-54.9%) were observed across the six lactating Holstein cows with Proteobacteria comprising the next most abundant group (1.9-3.5%).

Culture-dependent and culture-independent 16S rRNA methods were also applied with studies involving beef cattle [13-15]. Utilizing classical full length 16S rRNA gene sequence analysis a total of 1,906 OTUs (97% OTU designation) were identified from six cattle [14]. A core set of phyla were observed based on 24 OTUs comprised of 1,253 sequences (1.2% of OTUs obtained) with 1,348 OTUs found only in individual libraries. Seven phyla were found within six animals with three dominant taxonomic groups; Firmicutes, (62.8% of the OTUs), Bacteroidetes (29.5% of the OTUs) and Proteobacteria (4.4% of the OTUs). In another small study of beef cattle (n = 6) the DNA pyrosequencing method was applied to the comparison of the effects of three diets on ruminal (fistulated Jersey cows, n = 3) and fecal (Angus steers) bacterial assemblages [13]. Three diets (n = two cattle per diet, blocked by breed) in which of 0, 25, or 50% of the concentrate portion of the diet was replaced with dried distillers grains (DDGS) plus solubles were compared. Over 400 different bacterial species were detected that belonged to 56 separate genera from ruminal samples across all three diets. In all fecal samples, more than 540 different bacterial species were detected corresponding to 94 separate genera. The 25 most common genera that accounted for over 85% of the ruminal and fecal bacterial populations were identified. The Firmicutes: Bacteroidetes ratio tended to decrease as the proportion of DDGs increased.

In a much larger study involving 30 cattle distributed across geographically different locations and six different feeding operations (n = 5 cattle per operation) the DNA pyrosequencing method (633,877 high-quality reads) was used to assess fecal microbial community assemblages [15]. The majority of sequences were distributed across four phyla: Firmicutes (55.2%), Bacteroidetes (25.4%), Tenericutes (2.9%), and Proteobacteria (2.5%). Core taxa were observed across 5 different phyla: Actinobacteria (0.11% of all pyrotags; 0.67% of shared taxa), Bacteroidetes (5.7% of all; 13.3% of shared taxa), Cyanobacteria (0.08% of all; 3.33% of shared taxa), Firmicutes (17.5% of all; 73.3% of shared taxa), and Tenericutes (0.96% of all; 3.33% of shared taxa). Using sequence-based clustering and taxonomic analyses, less variability was observed within a particular management practice/location than among different management practices. Animal feeding operations seemed to influence bovine fecal bacterial communities at the phylum and family taxonomic levels much more so than geographic location of the feedlot. Lastly, overall bacterial community composition seemed to be strongly influenced by fecal starch concentrations. The most responsive phyla to diet were Bacteroidetes, Firmicutes and Proteobacteria. The relative abundance of Bacteroidetes increased with increasing fecal starch concentration, whereas, the abundance of Firmicutes decreased with increasing fecal starch concentrations.

In the present study, we used the barcode DNA pyrosequencing technique to evaluate the influence of five beef cattle diets on fecal microbial assemblages. The diets consisted of a traditional diet feed beef cattle in the Southern High Plains of Texas-Con (steam-flaked corn or 0% DG), and four diets containing different percentages of DGs in the dietary dry matter; 10 C (10% corn DG), 5S (5% sorghum DG), 10S (10% sorghum DG), and 15S (15% sorghum DG). The barcoded DNA pyrosequencing method was used to generate 16S OTUs dataset. The 16S OTUs dataset was assigned to various taxonomic classes and each phylogenetic level was analyzed using a variety of statistical tests including UniFrac procedures, hierarchal cluster analysis, distance based redundancy analysis (dbRDA), and One-way ANOVA to test the influence of dietary treatments on microbial populations. We describe significant changes in microbial community structure and diversity that is influenced by these different DGs diets.

## Results

### General DNA sequencing observations

**Figure 1:**
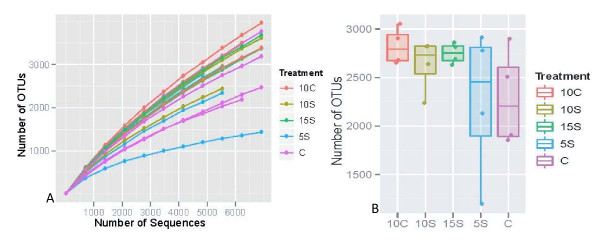
**Summary of diversity assessments based on operational taxonomic unit (OTUs) (3% divergence) for each sample**. **A**. Summary of rarefaction results based on operational taxonomic unit (OTUs) (3% divergence) for each sample. Rarefaction curves are displayed for each of the samples. CON = Control, 10 C = 10% Corn, 5S = 5% Sorghum, 10S = 10% Sorghum, 15S = 15% Sorghum. **B**. Summary of box plots revealing beta diversity associated with each treatment. The centroid (50%) and quantile (25 and 75%) values depicting the dispersion of OTUs associated with each dietary treatment. Dots indicate the OTUs associated with each animal. CON = Control, 10 C = 10% Corn, 5S = 5% Sorghum, 10S = 10% Sorghum, 15S = 15% Sorghum.

A total of 127,530 high quality 16S OTUs were utilized in the analysis (Table 1). The total number of high quality 16S OTUs recovered from each animal is listed in Table 1. The average number of OTUs returned for each diet was: CON, 6613; 10 C, 6836; 5S, 6042; 10S, 5977; and 15S, 6416. Rarefaction curves indicated that a high level of microbial diversity was obtained for subsequent analysis of dietary treatments (Figure 1a). In general, no treatment was associated with a loss of sample size for subsequent evaluation of populations across treatments. The total abundance observed for OTUs and their associated centroids distributed across treatments are indicated in box plots depicting beta diversity (Figure 1b). The highest abundance was observed in the 10 C diet followed closely by the 10S and 15S diets. The highest animal to animal variation was observed in the 5S diet followed closely by the control diet. In general, abundance ranges for the diets and their associated centroids were more tightly grouped with the 10S and 15S diets.

**Table 1:** Distribution of 16S OTUs amongst beef cattle fed wet DG

Treatment	Animal ID	No 16S OTUs
5S	123	5444

5S	140	6187

5S	147	5040

5S	255	7498

10 C	196	7519

10 C	201	5631

10 C	203	6303

10 C	378	7889

10S	49	5126

10S	198	6967

10S	258	5777

10S	295	6036

15S	54	7236

15S	149	6295

15S	188	6682

15S	328	5450

Con	20	6257

Con	55	7050

Con	157	6564

Con	296	6579

The dietary treatment, animal ID, and no. of OTUs obtained per fecal grab from each animal

**Figure 2:**
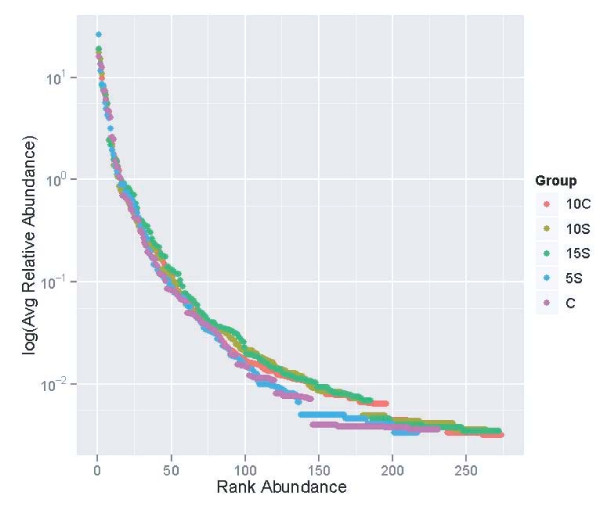
**Rank abundance curves for each treatment**. Each point represents the average relative abundance for a species, and species are ranked from most abundant to least abundant. CON = Control, 10 C = 10% Corn, 5S = 5% Sorghum, 10S = 10% Sorghum, 15S = 15% Sorghum.

The relationship among treatments is indicated in Whittaker plots (plotted as the log of the relative abundance vs. rank abundance) with each dot representing a species (Figure 2). The left and top of the graph indicate the presence of the most abundant OTUs with the bottom and right indicating the occurrence of rare OTUs. Each dot represents one species and the high steepness of the graph is indicative of unevenly distributed species. The lengths of the curves also indicate the occurrence of rare OTUs. The curves generally overlap one another in this analysis for all dietary treatments; thus, overall microbial diversity were similar.

### Influence of DGs on fecal microbiota-phyla

Four phyla were observed to have a response to dietary treatments (Additional file 1: Figure S1a-d). These are Synergistetes (*p *= 0.010), WS3 (*p *= 0.05), Actinobacteria (*p *= 0.06), and Spirochaetes (*p *= 0.06).

**Figure 3:**
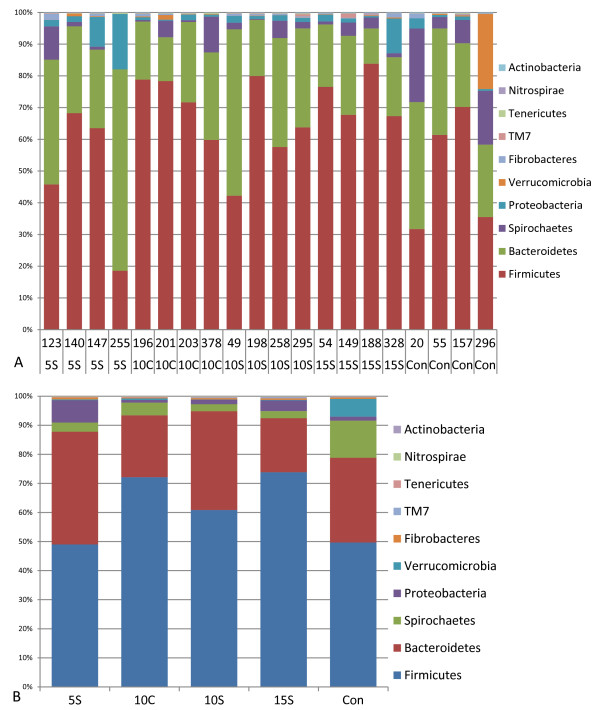
**Distributions of phyla**. **A**. The distribution of major phyla (≥ 99.5% abundance) based on bacterial counts among 20 beef cattle feed five diets. **B**. Distribution of the most abundant phyla averaged across the dietary treatments. CON = Control, 10 C = 10% Corn, 5S = 5% Sorghum, 10S = 10% Sorghum, 15S = 15% Sorghum.

A total of 24 phyla were observed distributed amongst all beef cattle on all diets (Figure 3a and Additional file 2: Figure S2). These are listed in order of average abundance and with their respective ranges (only the top ten abundances and ranges shown): Firmicutes (61%, 19-83%), Bacteroidetes (28%, 11-63%), Spirochaetes (5%, 0.0-23%), Proteobacteria (3.03%, 0.34-17.5%), Verrucomicrobia (1.43%,%,0.0-23.6%), Fibrobacteres (0.51%, 0.0-1.95%), TM7 (0.16%, 0.0-1.32%), Tenericutes (0.15%, 0.0-0.35%), Nitrospirae (0.11%, 0.03-0.22%), Actinobacteria (0.09%, 0.0-0.24%), and Fusobacteria (0.0863%, 0.0166-0.3813%). Chlamydiae, Cyanobacteria, Planctomycetes, Synergistetes, Lentisphaerae, Acidobacteria, Elusimicrobia, Chlorobi, WS3, Deinococcus-Thermus, Chloroflexi, Gemmatimonadetes, and Deferribacteres were defined as low abundance phyla. Greater than 99.4% of total bacterial abundance was observed in the first 10 phyla, with several remaining phyla represented by 5 or less members. The abundance levels of the top ten phyla averaged based on dietary treatment are presented in Figure 3b. A higher relative abundance of Firmicutes was observed when compared to the relative abundance level of Bacteroidetes for DGs diets that contain 10% or more DG supplement vs. the CON and 5S diets. However, significant differences were not observed among dietary treatments for abundances of Firmicutes (*p *= 0.11) and Bacteroidetes (*p *= 0.13) (Additional file 3: Figure S3a and S3b, respectively and Additional file 4: Table S1, Additional file 5: Table S2, respectively). A matched pair comparison evaluation of the abundances of Firmicutes to Bacteroidetes to one another yielded a non-significant response (Additional file 3: Figure S3c). A core set of six phyla were observed in all animals regardless of dietary treatment, and they were; Firmicutes, Bacteroidetes, Proteobacteria, Tenericutes, Nitrospirae, and Fusobacteria. With the exception of one animal (255) that lacked Spirochaetes, seven phyla would have been observed.

### Distribution of bacterial class, order and families by treatment

**Figure 4:**
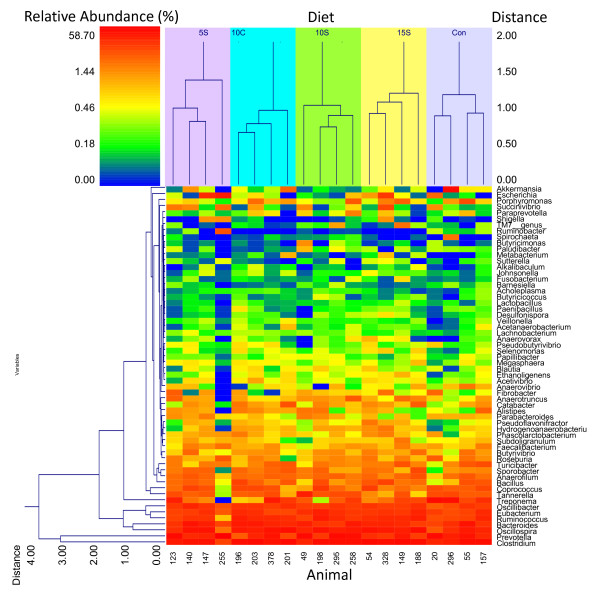
**Influence of wet DG diets on beef cattle fecal microbiota on the top 60 most abundant genera (representing ≥ 98% of the observed community)**. CON = Control, 10 C = 10% Corn, 5S = 5% Sorghum, 10S = 10% Sorghum, 15S = 15% Sorghum.

The response of the most abundant bacteria at the phylogenetic levels of class, order and family is revealed in a series of heat maps (Additional file 6: Figure S4) and, for further clarification, (Additional file 7: Figure S5a and b) in abundance plots showing both the individual animal response to diet and the averaged response to diet. For clarity and visualization purposes only the top 50 bacterial orders (Additional file 8: Figure S6) and the top 60 bacterial families (Additional file 9: Figure S7) are presented in heat maps. For corresponding abundance plots, the cutoffs are at the 97-99% abundance levels and orders and families are presented (Additional file 10: Figure S8a and b; Additional file 11: Figure S9a and b, respectively). With respect to abundance levels of Clostridia, Bacteroidia, and Gammaproteobacteria, animal 255 microbial community was the most disparate from all the other animals. The relative abundance of Clostridia was substantially lower and the relative abundance of Bacteroidia and Gammaproteobacteria were greater (Additional file 7: Figure S5a and b). This effect is expressed at the phylogenetic level of bacterial orders with lower Clostridiales and greater Bacteroidales and Enterobacteriales (Additional file 10: Figure S8a and b) down to the level of families with lower abundances of Ruminococcaceae and Clostridiaceae and greater levels of Prevotella (Additional file 11: Figure S9a and b). Other animals appeared to be variable with respect to one or two other taxa such as number 20, 123, and 296 when viewing patterns observed on the heat maps (e.g., Figure 4 and Additional file 9: Figure S7).

### Influence of DGs on fecal microbiota-genera

A total of 937 bacterial species were observed distributed among 446 genera across all fecal samples (data not shown). A double hierarchal dendrogram was constructed using the UPGMA clustering method and Manhattan distance method with no scaling (NCSS 2007, Kaysville, UT). The influence of DG diets on the fecal microbiome was apparent from double hierarchal cluster analysis on the top 60 most abundant genera (≥ 97.5% of total bacterial genera observed) and clustered by dietary treatment (Figure 4). With respect to diets, the least apparent phylogenetic distance (based on 16S OTUs distance) observed within the top cluster was with the 10 C diet (suggesting greatest similarity) and the most was with the 5S diets (most diverse). *Prevotella *and *Clostridium *occurred together in their own separate cluster, whereas *Oscillospira, Bacteroides, Ruminococcus, Eubacterium*, and *Oscillibacter *resided in the next most distant cluster. The other 53 genera cohabited in another main cluster. For animal 255 the microbial community seemed to be most unlike the other animals and this was apparently a result of a high relative abundance of Bacteroidetes and a low relative abundance of Firmicutes (Figure 3a). The average abundance by treatment of the top 60 genera (depicted in heatmap, Figure 4) and the response of taxa to diet (influenced by *p *< 0.10 or significantly affected by *p *< 0.05) are presented in Additional file 12: Table S3. In brief, those taxa that had a treatment response were: *Clostridium, Ruminococcus, Oscillibacter, Tannerella, Parabacteroides, Hydrogenoanaerobacterium, Pseudoflavonifractor, Acetivibrio, Ethanoligenens, Selenomonas, Desulfonispora*, and *Barnesiella*.

The top 80 species comprised approximately 91% of the total abundance observed (Additional file 13: Table S4) and the following also show a significant response to treatment as detailed above. These are: *Clostridium *sp., *Tannerella *sp., *Pseudoflavonifractor capillosus, Catabacter *sp., *Hydrogenoanaerobacterium saccharovorans, Ruminococcus bromii*, and *Parabacteroides merdae*.

**Figure 5:**
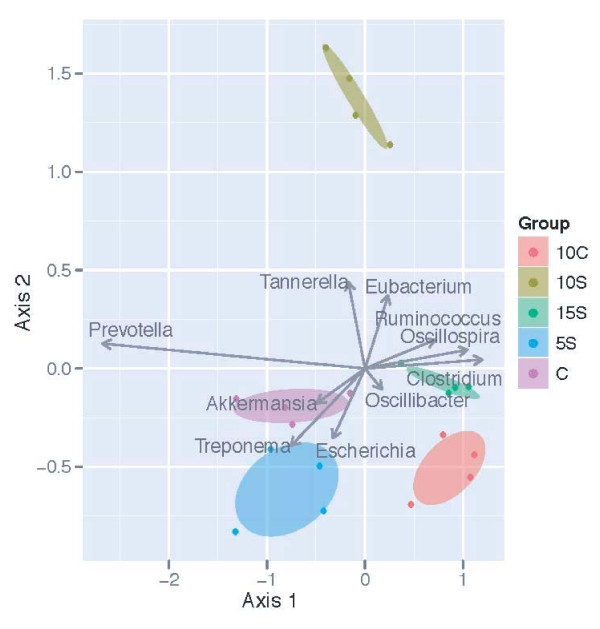
**Biplot of the dbRDA results when apparent phylogenetic distances (16S OTUs) among samples were measured using the weighted UniFrac distance measure**. Ellipses represent the 95% confidence interval around group centroids. Arrows indicate the contribution of individual taxa to the dbRDA axes, and only those taxa with the largest contributions are shown. In dbRDA the axis explains variation while being constrained to account for group differences (or, while being forced to illustrate how groups differ). CON = Control, 10 C = 10% Corn, 5S = 5% Sorghum, 10S = 10% Sorghum, 15S = 15% Sorghum.

**Figure 6:**
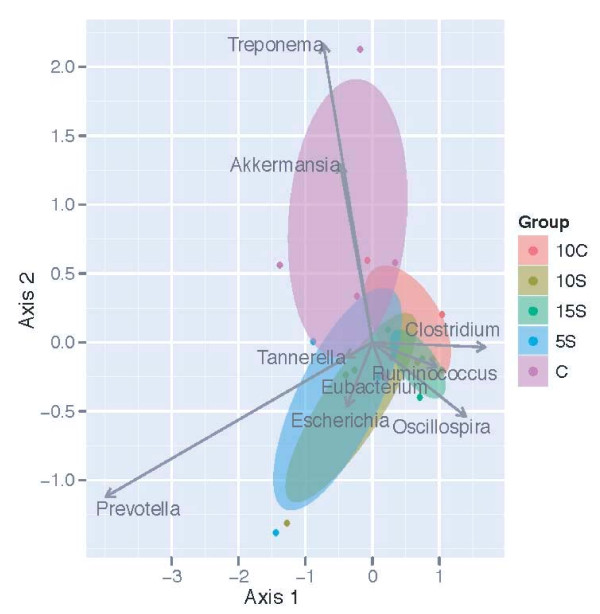
**Biplot of the dbRDA results when apparent phylogenetic distances (16S OTUs) among samples were measured using the unweighted UniFrac distance measure**. Ellipses represent the 95% confidence interval around group centroids. Arrows indicate the contribution of individual taxa to the dbRDA axes, and only those taxa with the largest contributions are shown. In dbRDA the axis explains variation while being constrained to account for group differences (or, while being forced to illustrate how groups differ). CON = Control, 10 C = 10% Corn, 5S = 5% Sorghum, 10S = 10% Sorghum, 15S = 15% Sorghum.

A biplot based on dbRDA using the unweighted UniFrac method identified taxa (Figure 5) that were significantly affected by diets, *p *= 0.043 (Table 2). Taxa most influenced by diet listed alphabetically were: *Akkermansia, Clostridium, Escherichia, Eubacterium, Oscillibacter, Oscillospira, Prevotella, Ruminococcus, Tannerella*, and *Treponema*. In Figure 5 the length and direction of the arrow (vector) with respect to diets indicates their relative positive or negative relationship to that diet. The ellipses around the animals represent the 95% confidence level, and their distance from one another reflects how closely or distantly the dietary effects are related to one another. It can be seen that *Akkermansia, Escherichia*, and *Treponema *were positively influenced by the 5S and CON diets, whereas the 10 C is situated to the lower right hand side of the figure indicating a weak response from *Oscillibacter*. The fecal community associated with the10S dietary treatment was least similar to the other fecal communities. A moderate influence of the 10S was observed for *Eubacterium *and *Tannerella*, whereas the 15S diet was near the point source eliciting a response from *Clostridium *and *Oscillospira*. The relative abundance of *Prevotella *seems to be positively influenced by the 5S and CON treatments since these diets are located on the lower axis 1. When analyzed using weighted UniFrac procedure a significant (*p *= 0.048) but slightly different result was observed regarding the influence of diets on microbial assemblages (Table 3). It can be seen that *Akkermansia *and *Treponema *relative abundance were positively influenced by the CON diet, whereas, *Escherichia *was orientated at nearly 180° from these two taxa, and was more abundant in the 5S and 15S diets (Figure 6). *Eubacterium *also had a similar response. *Prevotella *was oriented to the bottom left hand side of the figure, but it was much more in alignment with *Escherichia*.

**Table 2:** Results of an ANOVA like simulation test for the effects of treatment on the microbiome when distances among samples are measured using the unweighted UniFrac distance measure

	Df	Var	F	N.Perm	*P *(> F)
Treatment	4	0.38	1.51	999	0.043

Residual	15	0.94			

**Table 3:** Results of an ANOVA like simulation test for the effects of treatment on the when distances among samples are measured using the weighted UniFrac distance measure

	Df	Var	F	N.Perm	*P *(> F)
Treatment	4	1.29	1.11	999	0.048

Residual	15	4.35			

## Discussion

### Influence of distillers grain diets

Deep sequencing of 20 individual fecal samples from cattle fed five different diets (n = 4 per diet) provides a detailed view of the beef cattle fecal microbiome. The barcoded DNA pyrosequencing method yielded 127,530 high quality reads for microbiome comparison. We detected a core set of six bacterial phyla distributed across all animal fecal samples from all diets. In addition, we identified a total of 24 phyla distributed across a number of the fecal samples associated with the various diets that encompass 937 bacterial species distributed across 446 genera. We identified four phyla that were responsive to dietary treatments. These were Synergistetes (*p *= 0.01), WS3 (*p *= 0.05), Actinobacteria (*p *= 0.06), and Spirochaetes (*p *= 0.06). We also documented 12 genera and 7 species that responded to dietary treatments.

It can be difficult to make comparisons across these various cattle fecal studies since they have employed a variety of 16S rRNA-based sequencing strategies (choice of sequencing primers/sites and thus the type of phylogenetic information that can be extracted), the number and type of cattle employed in the studies and the types of diets and management practices associated with these diets. Short read lengths and potential biases in evenness (how many of each group) due to primer and template mismatches can result in pyro-sequencing artifacts that potentially affect taxonomic assignment and richness estimates [16]. This is especially so with respect to rare OTUs. Questions have also been posed and examined regarding the influence of geographical location, climatic conditions, and other localized environmental variables on cattle fecal microbial community structure [15]. Animal to animal variation was noted in fecal microbial diversity among beef cattle after controlling for location, climate, animal genetics, and diet [14]. Both the number and relative abundance of phyla we observed agree more closely with the distribution of phyla observed in the Shanks et al. [15] study than in the Callaway et al. study [13]. This could have been due to the number of cattle in the study (n = 30 vs. n = 6) or the size of the 16S OTUs dataset that was assembled (633,877 high-quality sequences). Both pyrosequencing studies [13,15] employed different primer locations and different read lengths to generate their datasets. The V6 region was specifically targeted in the Shanks study and used short read lengths (51 to 81 bases), whereas that of Callaway targeted the V4-V6 region (~500 bp region). Thus, of the studies described in detail [10,13-15], our results generally agree more closely with the findings of Shanks and Durso, despite using the methodology described by Dowd [10] and employed by Callaway [13]. One possible explanation is that our choice of primers targeted the V1 through V3 region of the 16S rRNA gene whereas the primer set utilized in the Callaway study used the V4 to V6 region to assess phylogenetic information. Another difference is that all of the cattle in the Dowd study [10] were lactating Holstein dairy cows and for the Callaway study [13] they were Jersey dairy cows and Angus steers.

A number of taxa appear to fluctuate in response to diets. Two taxa, *Ruminococcaceae *and *Prevotella *spp., had distinct patterns in response to dietary treatments, whereas, the majority of 512 taxa identified did not fluctuate across different dietary practices [15]. Other taxa identified in this study as being influenced by dietary treatment based on the UniFrac procedure were; *Akkermansia, Clostridium, Escherichia, Eubacterium, Oscillibacter, Oscillospira, Prevotella, Ruminococcus, Tannerella*, and *Treponema*. Two of these, *Prevotella *and *Ruminococcus*, were among those identified by Shanks [15]. We noted the presence of phyla in our study that were also present in the massive DNA pyrosequencing study of Shanks et al., [15] such as Actinobacteria, Spirochaetes, Verrucomicrobia, Cyanobacteria, Fibrobacteres, and Lentisphaerae. We also investigated the significance of the response of the dominant of phyla Firmicutes and Bacteroidetes to dietary treatments because these are highly abundant taxa and are thought to play a key role in energy capture. We also observed trends in Firmicutes and Bacteroidetes abundance as have others [13,15]; however, we could not identify a significant response of these phyla to diet.

The DG diets evaluated in these studies seemed to have a complex effect on fecal microbiota. Several of the procedures used in this study identified a common set of taxa that seem to be responsive to the influence of corn and sorghum DG diets vs. that of the traditional steam-flaked corn diet. Some of these taxa were identified in other studies as responsive to or seemingly influenced by starch content in the diet or the DG diet regardless of the differences in experimental protocols and animals (beef vs. dairy cattle). The presence of large animal to animal variation is noted in our study using a culture-independent method as well as in a culture dependent approach by Durso et al. [14]. However, the importance of a core set of taxa associated with the cattle bovine fecal microbiome is underscored by the fact that this core biome is observable regardless of the scale (ranging from thousands to hundreds of thousands of high quality reads) of sequencing efforts conducted across studies. It would appear that at least three phyla, Firmicutes, Bacteroidetes, and Proteobacteria comprise a core set of bacteria across all cattle types. Feeding corn- and sorghum-based DG in steam-flaked corn based diets resulted in significant shifts in the overall fecal microbial community structure ranging from phyla to genera. Ecological and evolutionary theory suggests that more diverse communities can make a greater contribution to ecosystem functioning [17,18]. If each species uses a slightly different resource and occupies a highly specific niche in the community, a more diverse microbiome should be able to, for example, more efficiently capture energy or be capable of capturing greater amounts of energy or possibly both. Clearly, bacterial communities in the bovine fecal environment are highly adapted and at the same time constrained by selection for existence in this environment. The presence of core taxa across all these studies implies that these microbes are involved in performing fundamental metabolic functions essential to the collective cattle microbiome. What the exact metabolic significance of these universal metabolic functions is, and if or how a shift in microbial populations (at the phylogenetic scale of the shifts observed across this microbiome) affects these universal metabolic functions remains to be determined. Daily weight gain and efficiency of weight gain (gain per unit of feed consumed) for the cattle in this experiment decreased linearly (*P *= 0.01) as the dietary concentration of sorghum DG increased; however, these measurements did not differ between corn and sorghum DG fed as 10% of the dietary DM [19]. The relationship between changes in cattle performance and alterations in the microbiome needs further study.

## Conclusions

This is, to our knowledge, the first study using this method to survey the fecal microbiome of beef cattle fed various concentrations of wet DG. Comparison of our results with other cattle DNA sequencing studies of beef and dairy cattle from a variety of geographical locations and different management practices identifies a core set of three phyla shared across all cattle. These three phyla in order of relative abundance are; Firmicutes, Bacteroidetes, and Proteobacteria. The presence of core taxa across all these studies implies that these microbes are involved in performing fundamental metabolic functions that are essential to the collective cattle microbiome. The presence of large animal-to-animal variation in cattle microbiome was noted in our study as well as by others.

## Methods

### Fecal collections and DNA Extraction

The animal feeding trial was approved by the Texas Tech University Animal Care and Use Committee (approved protocol number 0365-09). Details of the experimental design, location, animal management, and dietary chemical composition, are described in detail as Exp. 1 of Vasconcelos et al. [19]. A feeding trial employing five dietary treatments (20 cattle, n = 4 per diet) was conducted at the Texas Tech University Burnett Center near New Deal, TX. Two hundred crossbred beef steers (initial body weight of 404 ± 7.34 kg) were used in a randomized complete block design with the five dietary treatments replicated in eight weight blocks (1 pen for each treatment within each block). Pens had concrete floors, and partially slatted floors and were 2.9 m wide × 5.6 m deep with 2.4 m of linear bunk space. Ingredient composition of the five treatment diets employed in the study is presented in Table 4. Diets consisted of a CON (steam-flaked corn or 0% DG), 10 C (10% corn-based DG), 5S (5% sorghum-based DG), 10S (10% sorghum-based DG), and 15S (15% sorghum-based DG). All diets are essentially isonitrogenous with a formulated crude protein value of 13.5% (analyzed values of samples collected from the feed bunks ranged from approximately 11.7 to 12.3% [19]. Cottonseed meal was present only in the control and 5S diets at a level of 5.86 and 1.97%, respectively, whereas, sorghum DG was present at 5.37, 10.70, and 15.97% amount and corn DG was present at 10.20% amount. Thus, cottonseed meal was present only in one of the DG dietary treatments (5S). Steam-flaked corn concentrations decreased in correspondence with increasing DG concentrations.

**Table 4:** Dietary composition of the control and wet distillers grain diets used in the Lubbock feeding trials (from Exp. 1 of Vasconcelos et al., [19])

	Treatment diets				
**Ingredient**	**0**	**S5%**	**S10%**	**S15%**	**C10%**

Steam-flaked corn	**75.40**	**73.90**	**70.67**	**65.73**	**71.04**

Cottonseed hulls	7.62	7.59	7.56	7.53	7.60

Cottonseed meal	**5.86**	**1.97**	**-**	**-**	**-**

Urea	1.01	1.01	0.77	0.25	0.53

Limestone	0.26	0.35	0.52	0.81	0.53

Fat	3.06	3.05	3.04	3.02	3.06

Molasses	4.25	4.23	4.22	4.19	4.24

Supplement	2.54	2.53	2.52	2.50	2.50

Wet sorghum distillers grain	**-**	**5.37**	**10.70**	**15.97**	**-**

Wet corn distillers grain	**-**	**-**	**-**	**-**	**10.20**

The sorghum DG used in the experiment was obtained from an ethanol plant in New Mexico and was a composite (dry matter basis) of 47.1% sorghum centrifuge wet cake (directly from the centrifuge), 18.4% syrup, and 34.5% corn DDG (dry matter basis). The corn DG was composed (dry matter basis) of approximately 65% centrifuge wet cake and 35% syrup. Both sources of DG were stored in plastic silo bags for the duration of the experiment. Fecal samples were obtained on the day of shipment of cattle to slaughter after 141 days of feeding. Fecal samples were collected from 20 beef cattle (as fecal grab samples, one per steer). Fecal grabs were stored in the gloves used to collect the sample at -20°C until further processing.

DNA was extracted using the QIAamp DNA Stool Mini Kit (Qiagen, Valencia, CA) according to the manufacturer's protocol. DNA was quantified using agarose gel electrophoresis.

### Pyrosequencing

DNA pyrosequencing analysis was according to the bacterial tag-encoded FLX 16S rRNA (bTEFAP) method originally described by Dowd et al. [10]. Using 1-step PCR of 30 cycles based upon 28 F-519R primers. Sequences were quality trimmed Q25, depleted of short reads < 150 bp, reads with ambiguous base calls, and reads with homopolymer stretches > 6 bp. Clustering and denoising were performed using USEARCH 4.0 (http://Drive5.com) along with removal of singletons. The number of operational taxonomic units (OTUs) was used as a measure of microbiome richness, with OTUs being defined based on 3% divergence. Organism abundance was expressed as a percentage of total sequences generated. Organisms representing less than 1% of populations in all samples were grouped as "other" in graphs (supplemental information) or not graphed at all.

### Data analysis

DNA barcoded pyrosequencing analysis was performed to detect 4,000 to 6,000 sequences per sample. The number of operational taxonomic units (OTUs) was used as a measure of microbiome richness, and OTUs were defined based on 3% divergence. Before analysis, rarefaction was used to standardize the number of OTUs to a constant number of sequences, thus facilitating comparisons among groups. Differences in the number of OTUs among animal diets were evaluated using an ANOVA (see Tables in manuscript and supplementary information). Here, each dietary treatment was analyzed separately. For multivariate analysis, the 16S OTUs distances among samples first were calculated using the unweighted (bacterial counts as 0 and 1 observations) UniFrac distance measure ([20], which measures the phylogenetic distances among samples. The weighted (actual abundance) UniFrac distance measure was used because it also considers the relative abundance of each OTU (16S rRNA read) when calculating phylogenetic distances. Principle coordinates analysis (PCoA) was used to display these differences in 2 dimensions, thereby facilitating an overall assessment of variability in the entire microbiome among samples. To test for multivariate differences among treatment groups, distance based redundancy analysis (dbRDA) [21] was used. In addition, the relative abundances of all genera were evaluated using an ANOVA. Here, relative abundances were transformed (*p*' = arcsine (√*p*)) before analysis, and analyses were conducted separately for each of the diets. As an initial screening evaluation, uncontrolled *p*-values were used to screen taxa. Data are illustrated in figures in the manuscript and supplementary information. Rarefaction curves and UniFrac distances were calculated using QIIME [22], and all other analyses were conducted in R [23], using the vegan [24] and labdsv [25] packages. Double hierarchal cluster analysis was conducted using NCSS 2007 software (NCSS, Kaysville, UT) and one-way ANOVA was also conducted using JMP9 software (JMP, SAS, Cary, NC).

## Authors' contributions

MG and NAC designed the feeding trial which was conducted by MG; WCR and NAC acquired the samples. SED, SBC and WCR performed sequence and bioinformatics analysis. WCR analyzed and interpreted the data, and drafted the article. All authors provide editorial content and have read and approved the final manuscript.

"The U.S. Department of Agriculture (USDA) prohibits discrimination in all its programs and activities on the basis of race, color, national origin, age disability, and where applicable, sex, marital status, famial status, parental status, religion, sexual orientation, genetic information, political beliefs, reprisal, or because all or part of an individual's income is derived from any public assistance program. (Not all prohibited bases apply to all programs.) Persons with disabilities who required alternative means for communication of program information (Braille, large print, audiotape, etc.) should contact USDA's TARGET Center at (202) 720-2600 (voice and TDD). To file a complaint of discrimination, write to USDA, Office of Civil Rights, 1400 Independence Avenue, S.W., Washington, D.C. 20250-9410, or call (800) 795-3272 (voice) or (202) 720-6382 (TDD). USDA is an equal opportunity provider and employer. Mention of trade names or commercial products in this article is solely for the purpose of providing specific information and does not imply recommendation or endorsement by the U.S. Department of Agriculture.

## Supplementary Material

Additional file 1 **Figure S1**. Evaluation of Bacteroidetes and Firmicutes relative abundance to the influence of dietary treatments, (**A**) One-way Analysis of Firmicutes by Treatment, (**B**) One-way Analysis of Bacteroidetes by Treatment, and (**C**) Matched pair comparisons testing the response of the ratio of abundances observed between Bacteroidetes and Firmicutes revealing no significant difference between and amongst treatments. Click here for file

Additional file 2 **Figure S2**. Evaluation of Phyla showing a response (significant < 0.05, or influenced < 0.1) to dietary treatments (**A**) Oneway analysis of Synergistetes by treatment, (**B**) Oneway analysis of WS3 by treatment, (**C**) Oneway analysis of Actinobacteria by treatment, (**D**) Oneway analysis of Spirochaetes by treatment. Click here for file

Additional file 3 **Figure S3**. Effect of wet DG's on Beef Cattle Fecal Microbiota. The influence on DDG's diets on beef cattle fecal microbiota relative abundance at the level of phyla is revealed by a hierarchal clustering double dendrogram (heatmap) based upon the relative abundance of 24 phyla. Click here for file

Additional file 4 **Table S1. A-C **Evaluation of Major Phyla for Response to Dietary treatments. Associated statistical tables for Additional file 3: Figure S2A-C. A One-way Analysis of Firmicutes by Treatment, B One-way Analysis of Bacteroidetes by Treatment, C Matched pair comparisons testing the response of the ratio of abundances observed between Bacteroidetes and Firmicutes. Click here for file

Additional file 5 **Table S2. A-D **Evaluation of Phyla showing a response (significant < 0.05, influenced < 0.1) to dietary treatments. Associated statistical tables for Additional file 1: Figure S1A-D. A Oneway Analysis of Synergistetes by Treatment, B Oneway Analysis of WS3 by Treatment, C Oneway Analysis of Actinobacteria by Treatment, D Oneway Analysis of Spirochaetes by Treatment. Click here for file

Additional file 6 **Figure S4**. Influence of DDG's diets on beef cattle fecal microbiota at the level of bacterial classes. Click here for file

Additional file 7 **Figure S5**. Influence of DDG's diets on beef cattle fecal microbiota at the level of bacterial families. Click here for file

Additional file 8 **Figure S6**. (**A**) Distribution of bacterial classes amongst diets and animals as revealed by heatmap. (**B**) Distribution of bacterial class's average across diets and animals. Click here for file

Additional file 9 **Figure S7**. Influence of DDG's diets on beef cattle fecal microbiota at the level of bacterial families. Click here for file

Additional file 10 **Figure S8**. (**A**) Distribution of bacterial orders (> 99% abundance) amongst diets and animals. (**B**) Distribution of bacterial orders (> 99% abundance) average across diets and animals. Click here for file

Additional file 11 **Figure S9**. (**A**) Distribution of the top (≥ 97% abundant) families observed amongst dietary treatments. (**B**) Distribution of the top (≥ 97% abundant) families averaged observed amongst dietary treatments. Click here for file

Additional file 12 **Table S3**. Average abundance of taxa by treatment. Taxa that showed a response to dietary treatment (see SEM and *P*-values). Click here for file

Additional file 13 **Table S4**. Average abundance of species by treatment. Species that showed a response to dietary treatment (see SEM and *P*-values). Click here for file
