# Supplementary figures and images for: Influence of wet distillers grains diets on beef cattle fecal bacterial community structure
Source: BMC Microbiol. 2012 Feb 24;12:25. doi: 10.1186/1471-2180-12-25 (PMC3305651; doi:10.1186/1471-2180-12-25)

## Slide 1
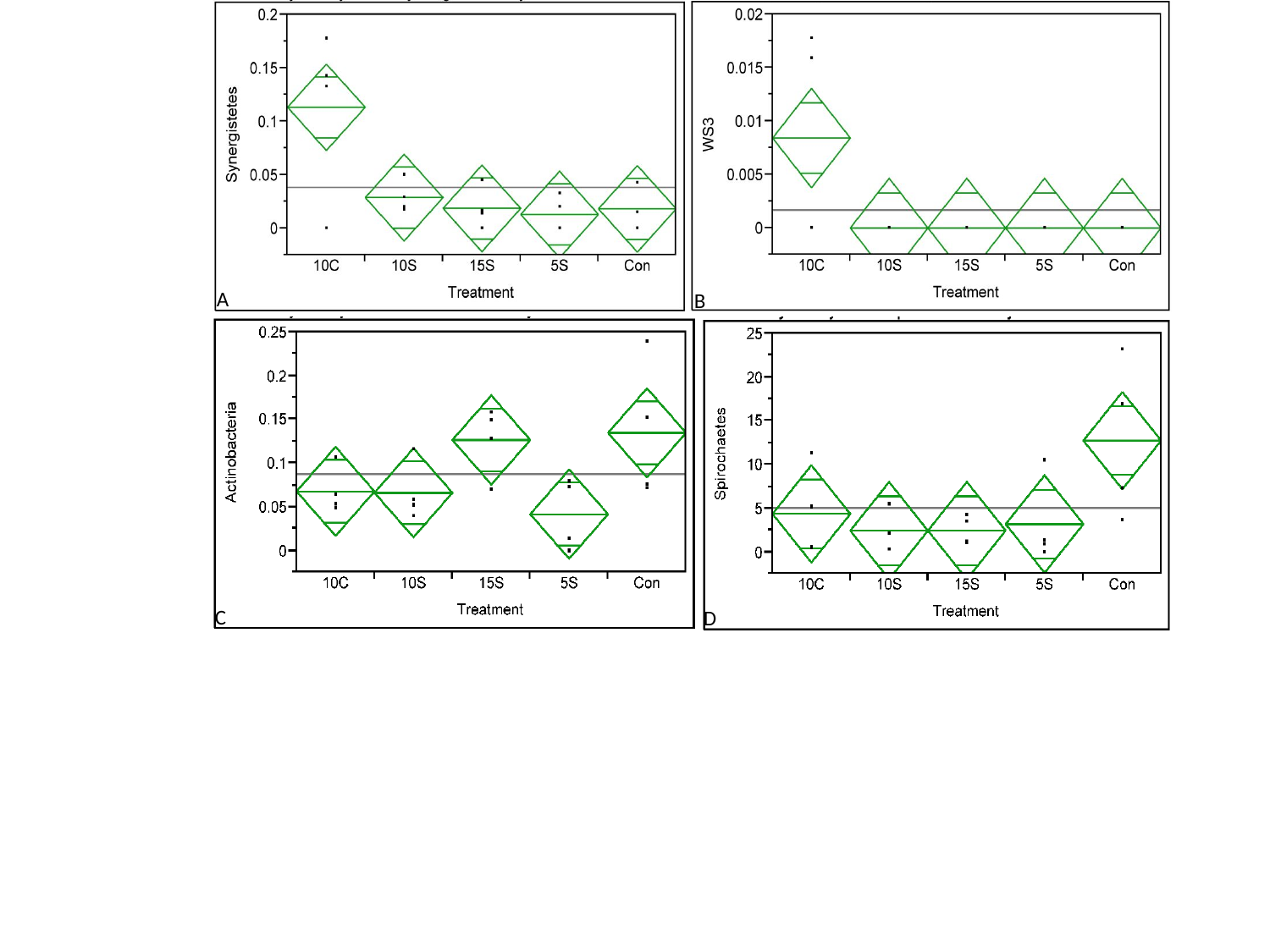

A
B
C
D

Supplement: Additional file 1 — Figure S1. Evaluation of Bacteroidetes and Firmicutes relative abundance to the influence of dietary treatments, (A) One-way Analysis of Firmicutes by Treatment, (B) One-way Analysis of Bacteroidetes by Treatment, and (C) Matched pair comparisons testing the response of the ratio of abundances observed between Bacteroidetes and Firmicutes revealing no significant difference between and amongst treatments. [file 1471-2180-12-25-S1.PPT]

## Slide 1
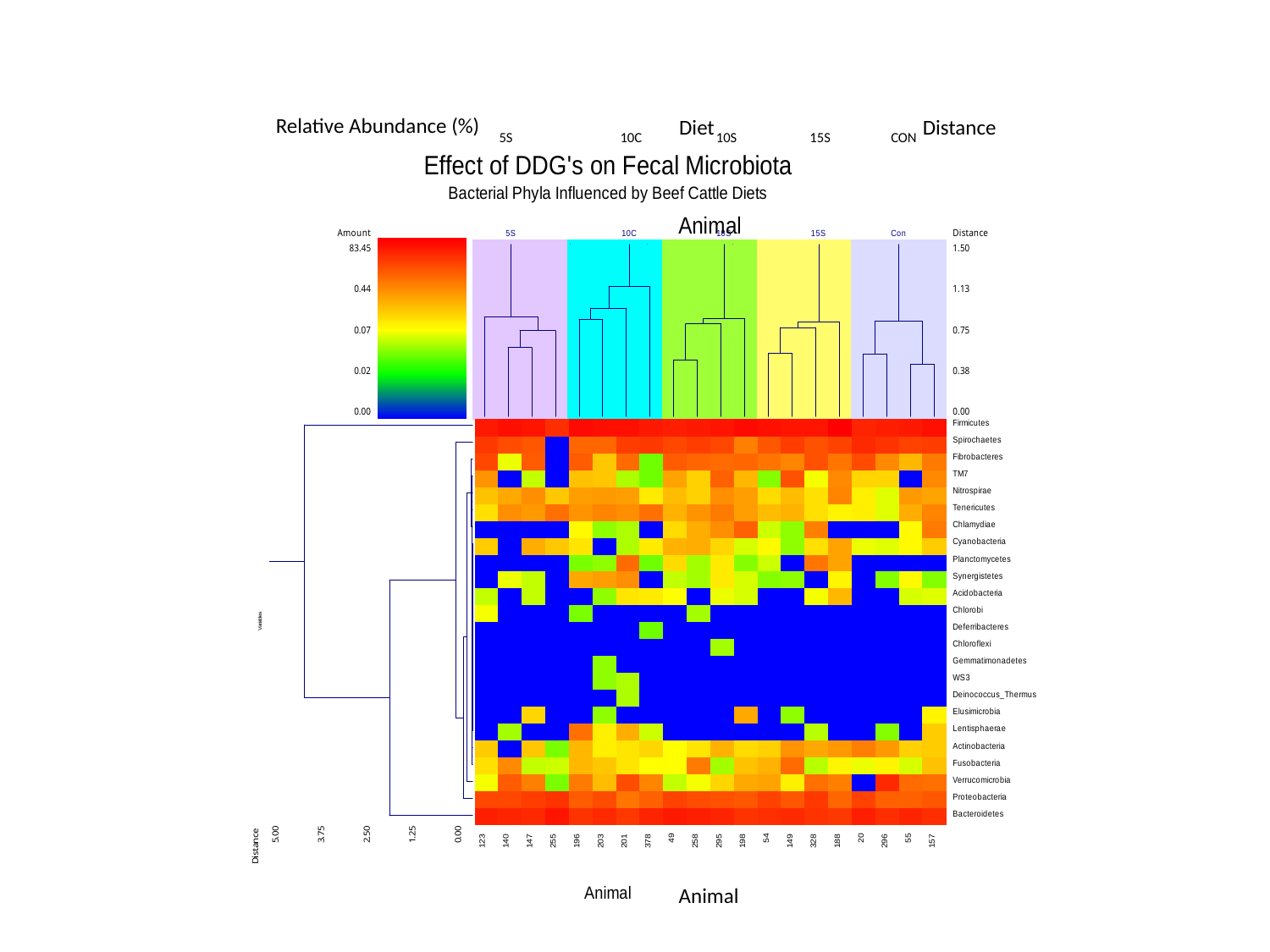

Relative Abundance (%)
Distance
Diet
5S
10C
10S
15S
CON
Animal

Supplement: Additional file 2 — Figure S2. Evaluation of Phyla showing a response (significant < 0.05, or influenced < 0.1) to dietary treatments (A) Oneway analysis of Synergistetes by treatment, (B) Oneway analysis of WS3 by treatment, (C) Oneway analysis of Actinobacteria by treatment, (D) Oneway analysis of Spirochaetes by treatment. [file 1471-2180-12-25-S2.PPT]

## Slide 1
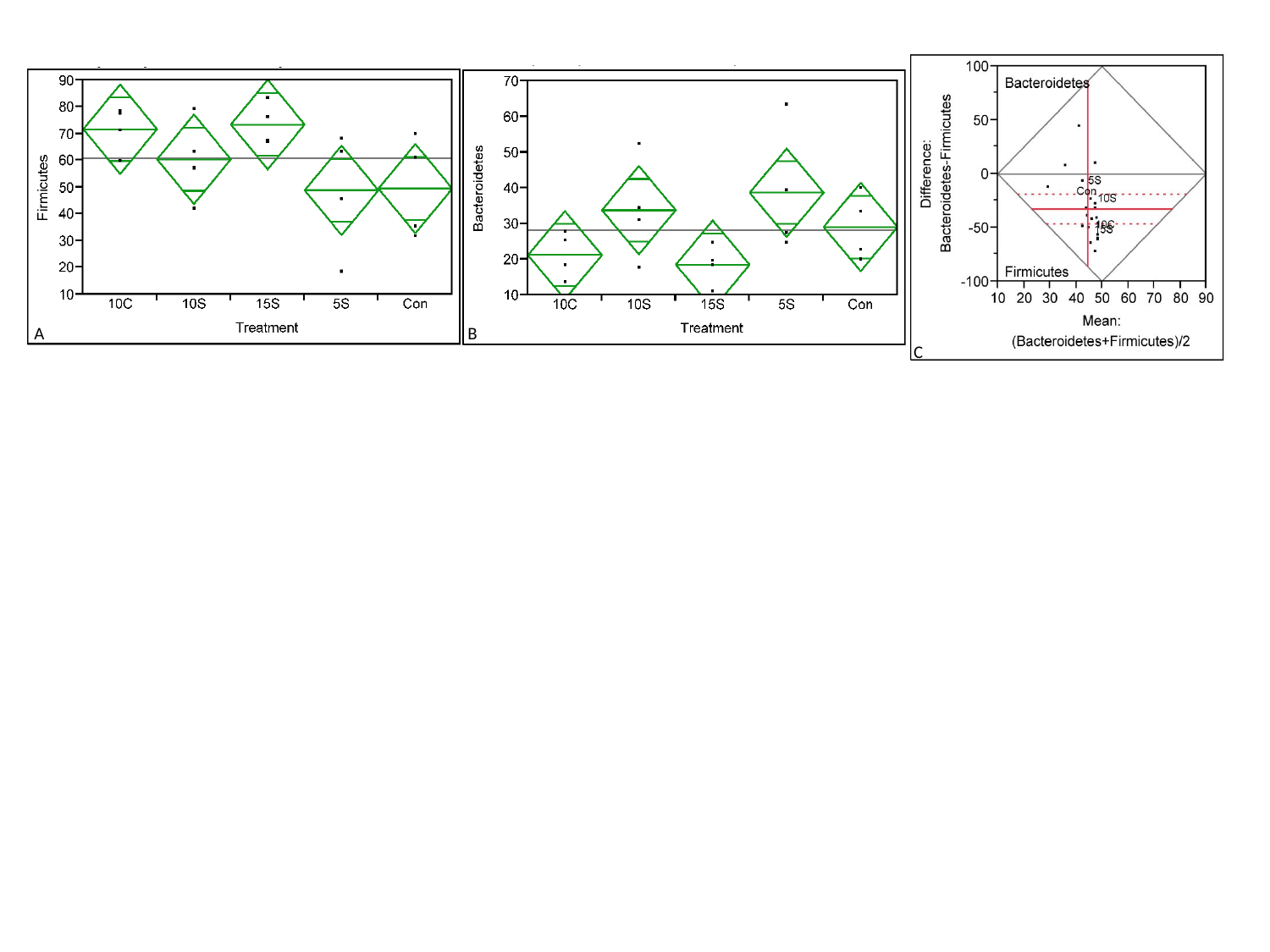

A
B
C

Supplement: Additional file 3 — Figure S3. Effect of wet DG's on Beef Cattle Fecal Microbiota. The influence on DDG's diets on beef cattle fecal microbiota relative abundance at the level of phyla is revealed by a hierarchal clustering double dendrogram (heatmap) based upon the relative abundance of 24 phyla. [file 1471-2180-12-25-S3.PPT]

## Slide 1
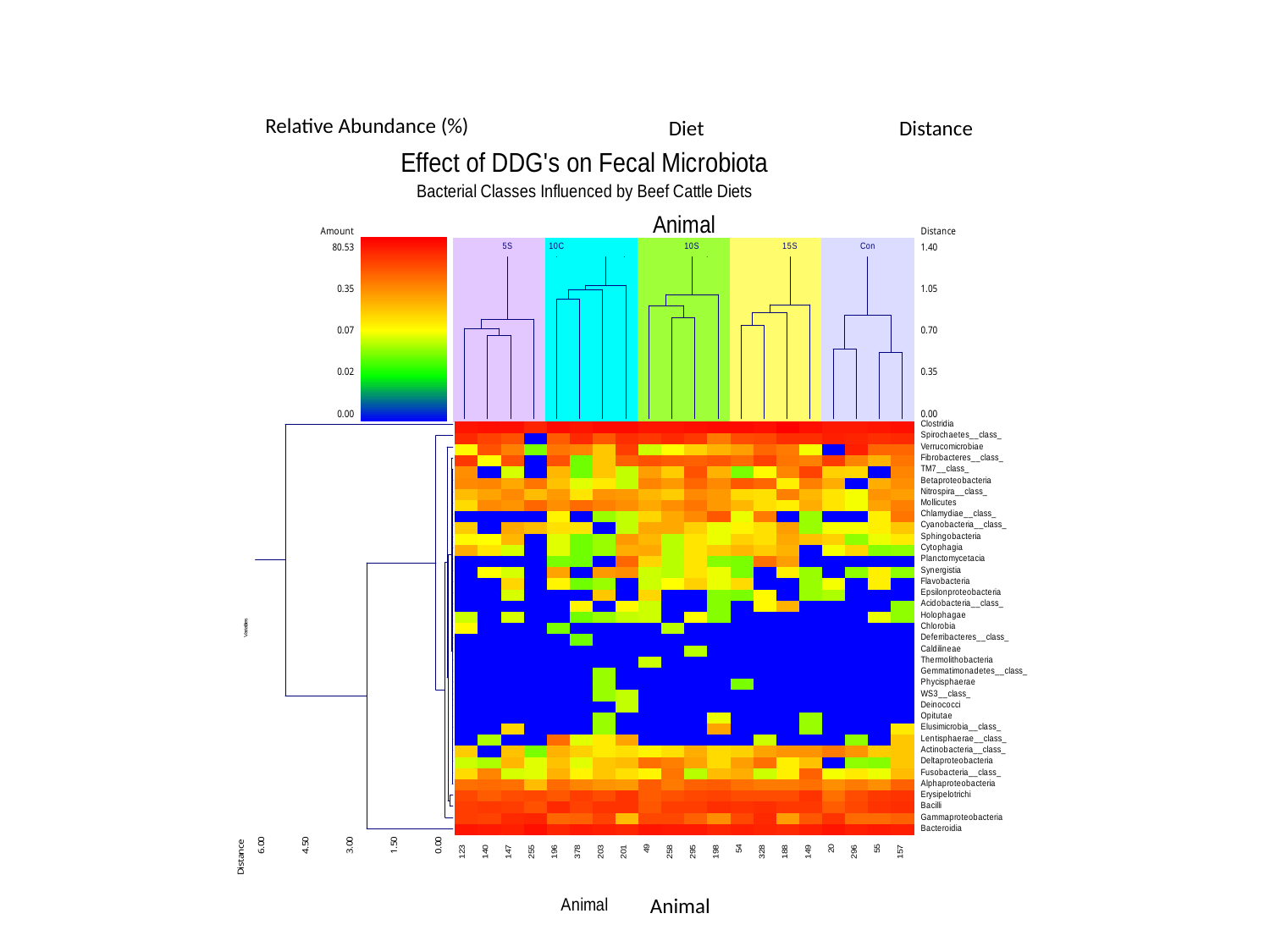

Relative Abundance (%)
Diet
Distance
Animal

Supplement: Additional file 6 — Figure S4. Influence of DDG's diets on beef cattle fecal microbiota at the level of bacterial classes. [file 1471-2180-12-25-S6.PPT]

## Slide 1
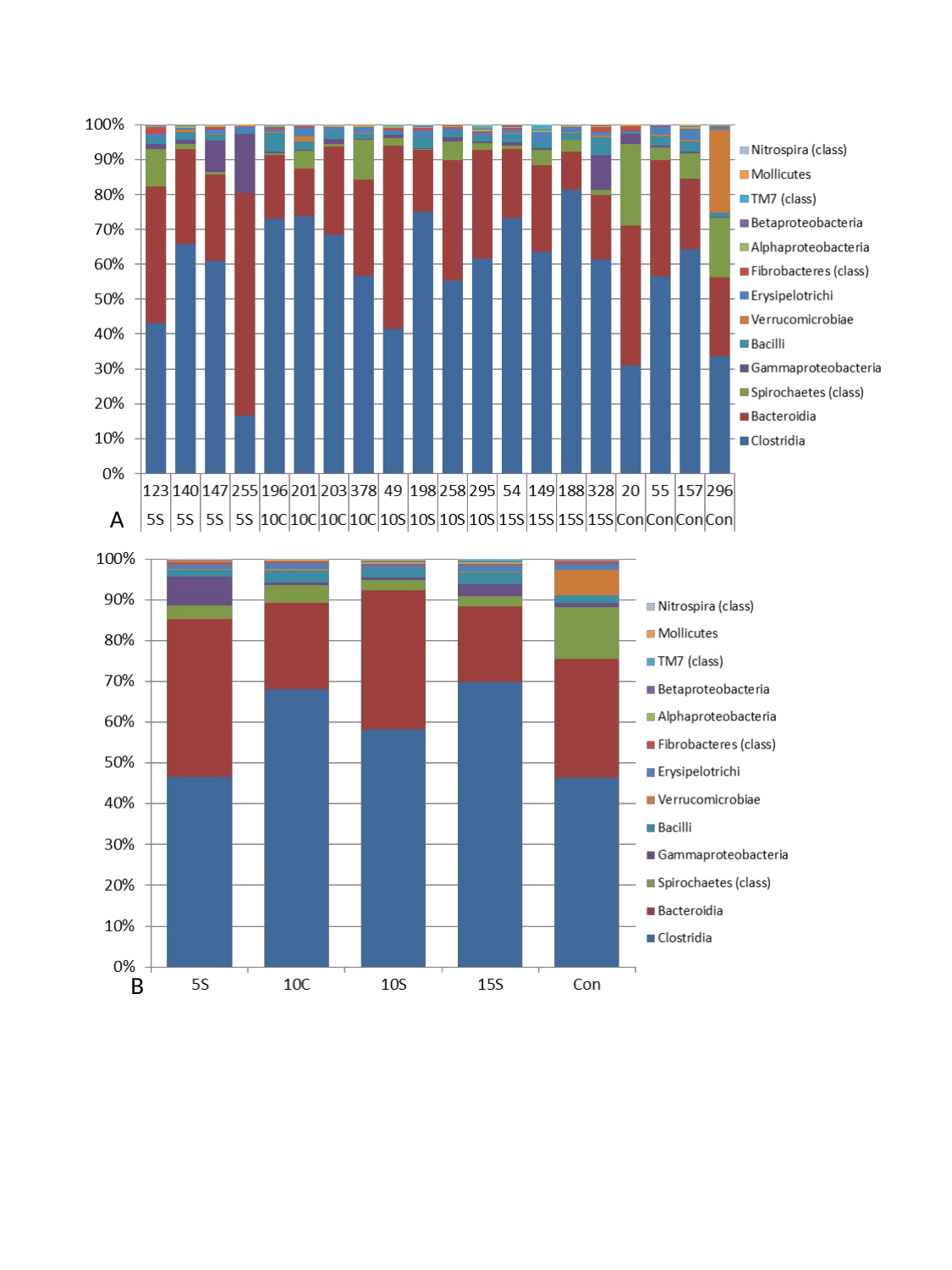

A
B

Supplement: Additional file 7 — Figure S5. Influence of DDG's diets on beef cattle fecal microbiota at the level of bacterial families. [file 1471-2180-12-25-S7.PPT]

## Slide 1
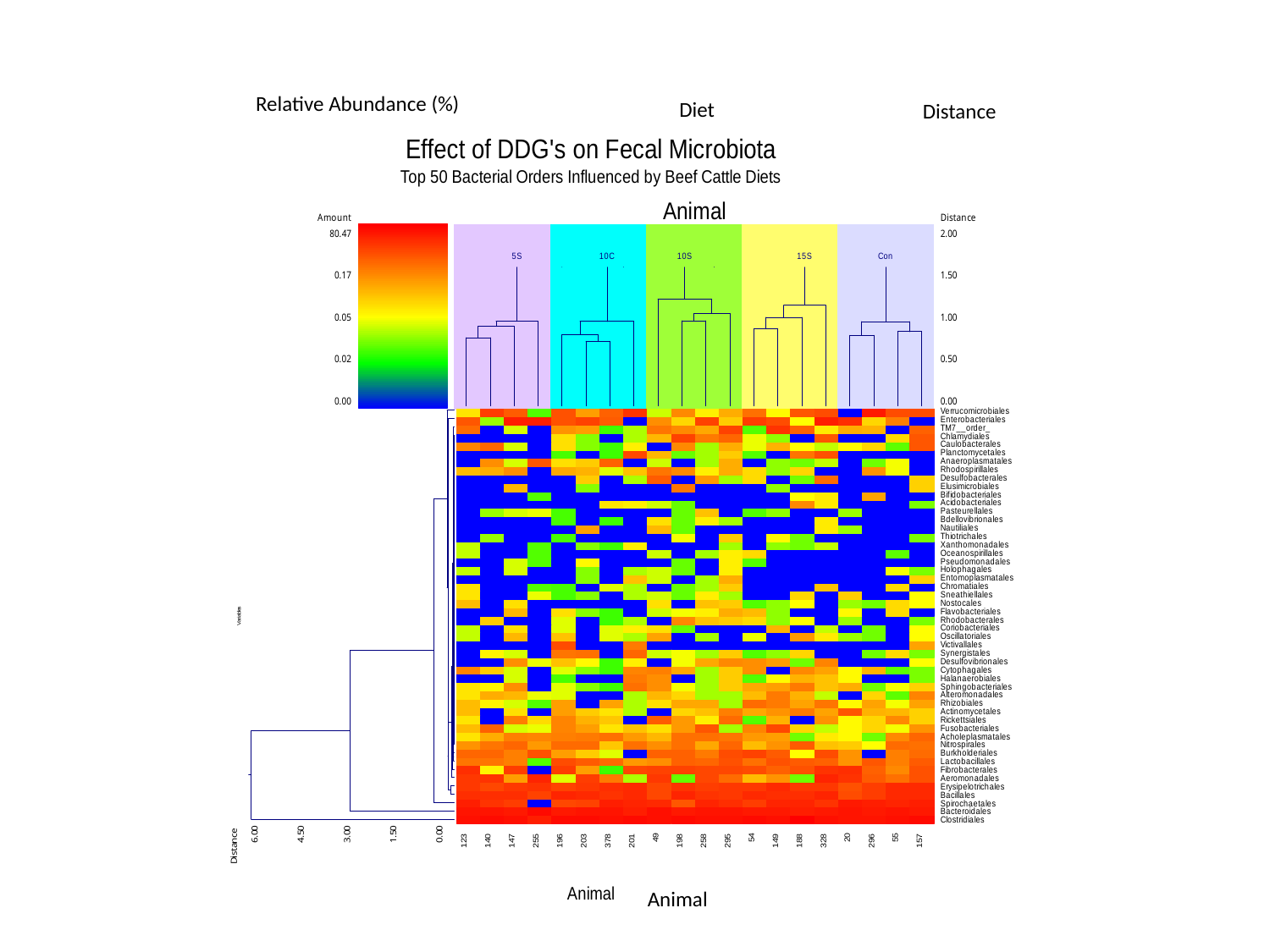

Relative Abundance (%)
Diet
Distance
Animal

Supplement: Additional file 8 — Figure S6. (A) Distribution of bacterial classes amongst diets and animals as revealed by heatmap. (B) Distribution of bacterial class's average across diets and animals. [file 1471-2180-12-25-S8.PPT]

## Slide 1
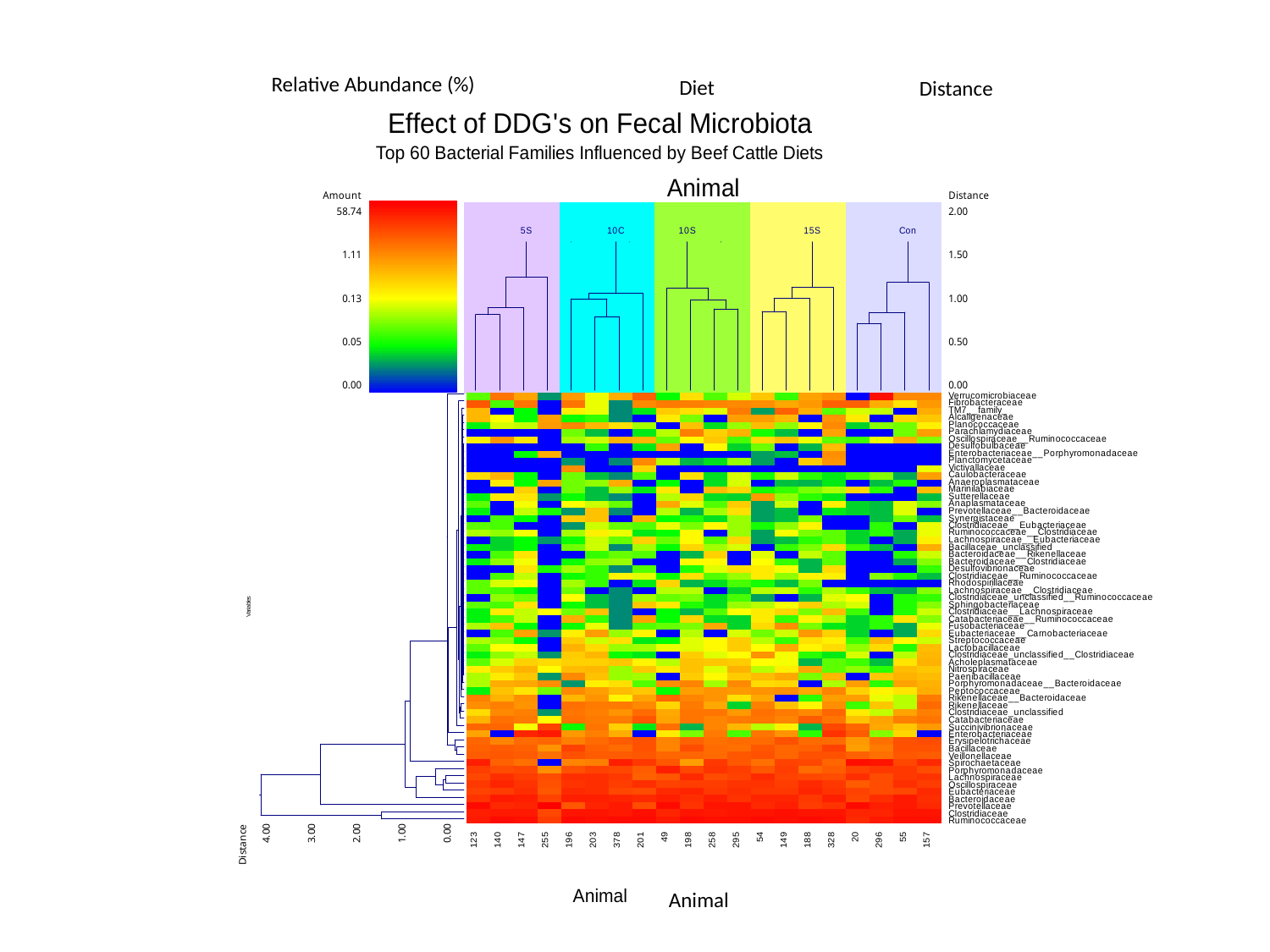

Relative Abundance (%)
Diet
Distance
Animal

Supplement: Additional file 9 — Figure S7. Influence of DDG's diets on beef cattle fecal microbiota at the level of bacterial families. [file 1471-2180-12-25-S9.PPT]

## Slide 1
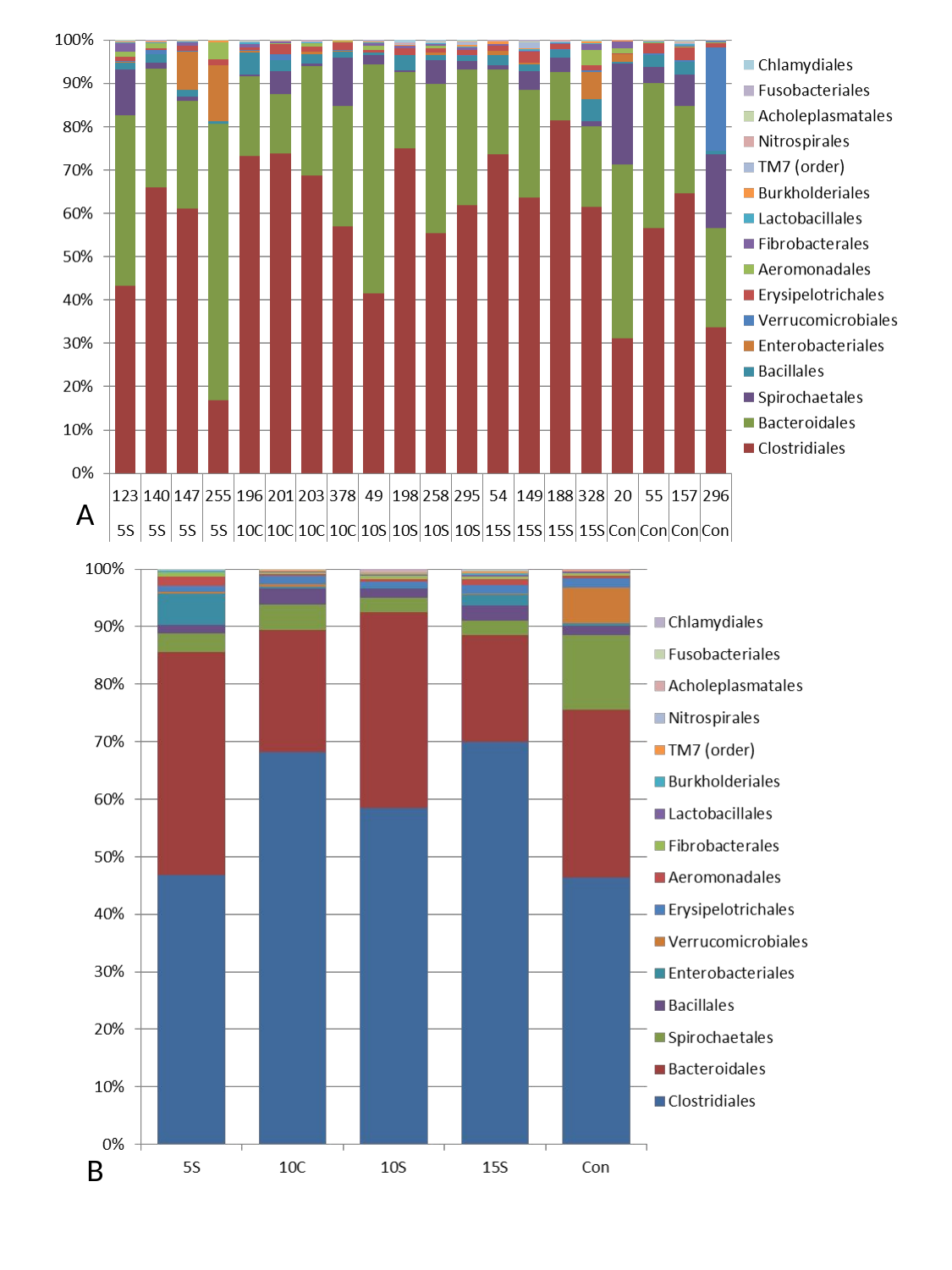

A
B

Supplement: Additional file 10 — Figure S8. (A) Distribution of bacterial orders (> 99% abundance) amongst diets and animals. (B) Distribution of bacterial orders (> 99% abundance) average across diets and animals. [file 1471-2180-12-25-S10.PPT]

## Slide 1
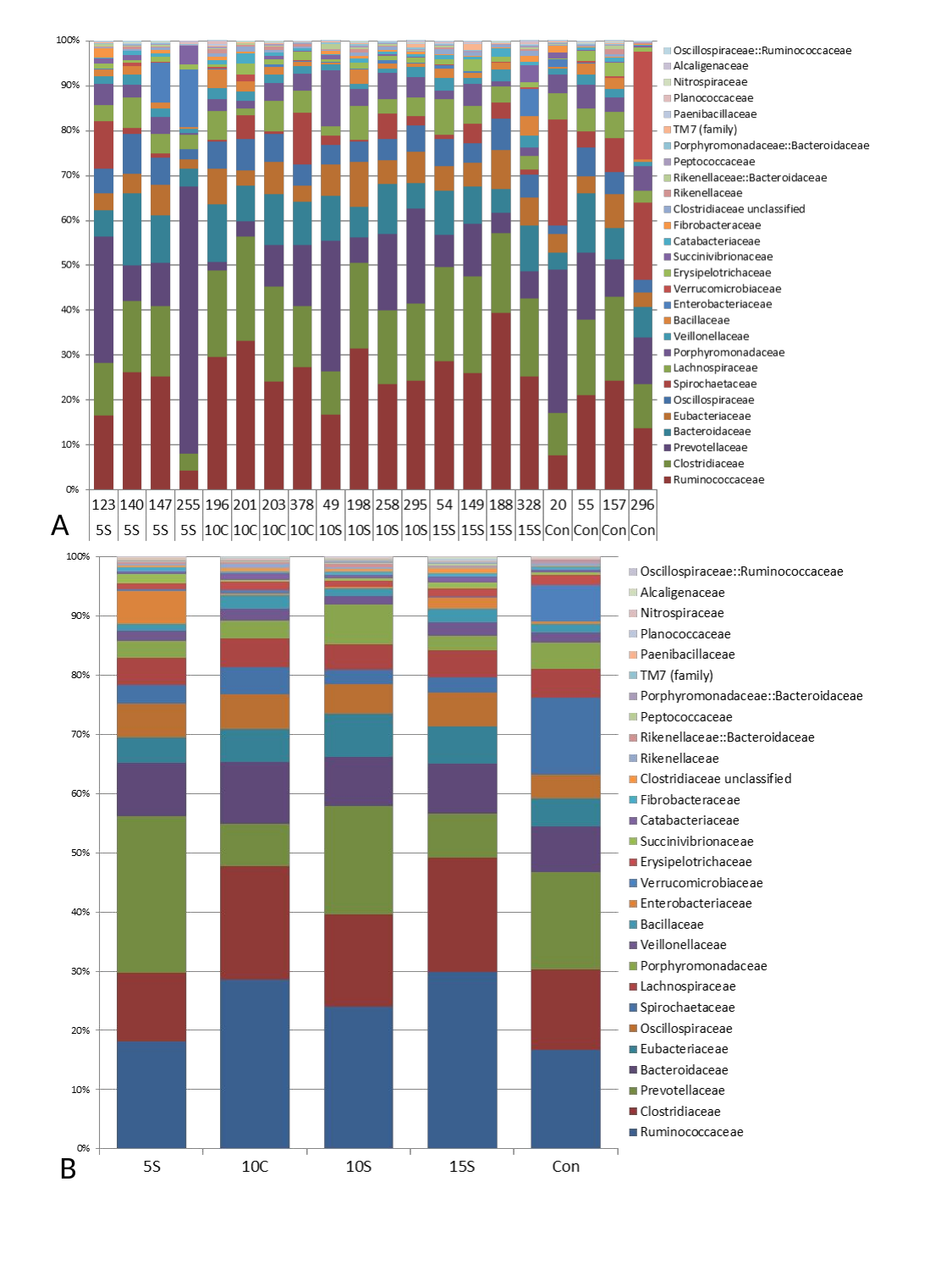

A
B

Supplement: Additional file 11 — Figure S9. (A) Distribution of the top (≥ 97% abundant) families observed amongst dietary treatments. (B) Distribution of the top (≥ 97% abundant) families averaged observed amongst dietary treatments. [file 1471-2180-12-25-S11.PPT]
