# Supplementary material for: Influence of wet distillers grains diets on beef cattle fecal bacterial community structure
Source: BMC Microbiol. 2012 Feb 24;12:25. doi: 10.1186/1471-2180-12-25 (PMC3305651; doi:10.1186/1471-2180-12-25)
Supplement: Additional file 4 — Table S1. A-C Evaluation of Major Phyla for Response to Dietary treatments. Associated statistical tables for Additional file 3: Figure S2A-C. A One-way Analysis of Firmicutes by Treatment, B One-way Analysis of Bacteroidetes by Treatment, C Matched pair comparisons testing the response of the ratio of abundances observed between Bacteroidetes and Firmicutes. [file 1471-2180-12-25-S4.DOC]

**Additional File 4, Table S1** Evaluation of Major Phyla for Response to Dietary treatments. Associated statistical tables for Fig S2-A-C.

A One-way Analysis of Firmicutes by Treatment

| Source | DF | Sum of Squares | Mean Square | F Ratio | Prob > F |
| --- | --- | --- | --- | --- | --- |
| Treatment | 4 | 2199.0919 | 549.773 | 2.2333 | 0.1143 |
| Error | 15 | 3692.5404 | 246.169 |  |  |
| C. Total | 19 | 5891.6323 |  |  |  |

B One-way Analysis of Bacteroidetes by Treatment

| Source | DF | Sum of Squares | Mean Square | F Ratio | Prob > F |
| --- | --- | --- | --- | --- | --- |
| Treatment | 4 | 1141.6574 | 285.414 | 2.1130 | 0.1297 |
| Error | 15 | 2026.1623 | 135.077 |  |  |
| C. Total | 19 | 3167.8197 |  |  |  |

C Matched pair comparisons testing the response of the ratio of abundances observed between Bacteroidetes and Firmicutes.

Matched Pairs - Difference: Bacteroidetes-Firmicutes

| Bacteroidetes | 28.2387 |  | t-Ratio | -4.96881 |
| --- | --- | --- | --- | --- |
| Firmicutes | 60.8629 |  | DF | 19 |
| Mean Difference | -32.624 |  | Prob > |t| | <.0001* |
| Std Error | 6.5658 |  | Prob > t | 1.0000 |
| Upper 95% | -18.882 |  | Prob < t | <.0001* |
| Lower 95% | -46.367 |  |  |  |
| N | 20 |  |  |  |
| Correlation | -0.8475 |  |  |  |

Across Groups

| Treatment | Count | Mean Difference | Mean Mean |
| --- | --- | --- | --- |
| 10C | 4 | -50.6 | 46.472 |
| 10S | 4 | -26.74 | 47.155 |
| 15S | 4 | -55.04 | 46.01 |
| 5S | 4 | -10.24 | 43.803 |
| Con | 4 | -20.5 | 39.315 |

| Test Across Groups | F Ratio | Prob>F |  |  |
| --- | --- | --- | --- | --- |
| Mean Difference | 2.1857 | 0.1201 | Within Pairs | Y Axis |
| Mean Mean | 2.2379 | 0.1137 | Among Pairs | X Axis |
