# Supplementary material for: Influence of wet distillers grains diets on beef cattle fecal bacterial community structure
Source: BMC Microbiol. 2012 Feb 24;12:25. doi: 10.1186/1471-2180-12-25 (PMC3305651; doi:10.1186/1471-2180-12-25)
Supplement: Additional file 5 — Table S2. A-D Evaluation of Phyla showing a response (significant < 0.05, influenced < 0.1) to dietary treatments. Associated statistical tables for Additional file 1: Figure S1A-D. A Oneway Analysis of Synergistetes by Treatment, B Oneway Analysis of WS3 by Treatment, C Oneway Analysis of Actinobacteria by Treatment, D Oneway Analysis of Spirochaetes by Treatment. [file 1471-2180-12-25-S5.DOC]

**Additional File 5, Table S2** Evaluation of Phyla showing a response (significant <0.05, influenced <0.1) to dietary treatments. Associated statistical tables for Fig S3A-D.

A Oneway Analysis of Synergistetes by Treatment

| Source | DF | Sum of Squares | Mean Square | F Ratio | Prob > F |
| --- | --- | --- | --- | --- | --- |
| Treatment | 4 | 0.02859855 | 0.007150 | 4.9525 | 0.0095* |
| Error | 15 | 0.02165456 | 0.001444 |  |  |
| C. Total | 19 | 0.05025311 |  |  |  |

B Oneway Analysis of WS3 by Treatment

| Source | DF | Sum of Squares | Mean Square | F Ratio | Prob > F |
| --- | --- | --- | --- | --- | --- |
| Treatment | 4 | 0.00022714 | 0.000057 | 2.9810 | 0.0537 |
| Error | 15 | 0.00028573 | 0.000019 |  |  |
| C. Total | 19 | 0.00051287 |  |  |  |

C Oneway Analysis of Actinobacteria by Treatment

| Source | DF | Sum of Squares | Mean Square | F Ratio | Prob > F |
| --- | --- | --- | --- | --- | --- |
| Treatment | 4 | 0.02685626 | 0.006714 | 2.9353 | 0.0562 |
| Error | 15 | 0.03431053 | 0.002287 |  |  |
| C. Total | 19 | 0.06116679 |  |  |  |

D Oneway Analysis of Spirochaetes by Treatment

| Source | DF | Sum of Squares | Mean Square | F Ratio | Prob > F |
| --- | --- | --- | --- | --- | --- |
| Treatment | 4 | 307.52626 | 76.8816 | 2.8136 | 0.0633 |
| Error | 15 | 409.86957 | 27.3246 |  |  |
| C. Total | 19 | 717.39583 |  |  |  |
