# Supplementary material for: Influence of wet distillers grains diets on beef cattle fecal bacterial community structure
Source: BMC Microbiol. 2012 Feb 24;12:25. doi: 10.1186/1471-2180-12-25 (PMC3305651; doi:10.1186/1471-2180-12-25)
Supplement: Additional file 12 — Table S3. Average abundance of taxa by treatment. Taxa that showed a response to dietary treatment (see SEM and P-values). [file 1471-2180-12-25-S12.DOC]

**Additional File 12, Table S3** Average abundance of taxa by treatment. Taxa that showed a response to dietary treatment (see SEM and P-values).

| **Genera by diet** | **5S** | **10C** | **10S** | **15S** | **Con** | **SEM** | ***P-*value** |
| --- | --- | --- | --- | --- | --- | --- | --- |
| Clostridium | 11.5019 | 18.7117 | 15.0634 | 18.9231 | 13.3810 | 2.0700 | 0.0876 |
| Prevotella | 25.8647 | 6.6604 | 17.2723 | 6.9424 | 15.8654 |  |  |
| Oscillospira | 8.3247 | 13.3542 | 10.8974 | 13.6214 | 7.3289 |  |  |
| Bacteroides | 8.6125 | 9.7578 | 7.9022 | 7.5353 | 7.3951 |  |  |
| Ruminococcus | 3.9826 | 8.1301 | 6.7411 | 8.3455 | 4.7295 | 1.1100 | 0.0457 |
| Eubacterium | 4.2202 | 5.4542 | 7.1435 | 6.1938 | 4.6107 |  |  |
| Oscillibacter | 5.6246 | 5.9067 | 4.8407 | 5.5662 | 4.0149 | 2.5700 | 0.0683 |
| Treponema | 3.1550 | 4.3178 | 2.3621 | 2.4249 | 12.4283 |  |  |
| Tannerella | 1.7367 | 1.7616 | 4.5731 | 1.0644 | 2.6041 | 0.8490 | 0.0818 |
| Coprococcus | 1.9530 | 2.5277 | 2.0855 | 1.9062 | 2.4727 |  |  |
| Bacillus | 1.2265 | 2.2137 | 1.3297 | 2.1590 | 1.3336 |  |  |
| Escherichia | 4.8842 | 0.1993 | 0.1691 | 1.5385 | 0.4118 |  |  |
| Akkermansia | 0.2788 | 0.5000 | 0.0475 | 0.1895 | 6.0776 |  |  |
| Anaerofilum | 1.3553 | 1.4776 | 1.3691 | 1.5635 | 1.3034 |  |  |
| Sporobacter | 1.1290 | 1.5997 | 1.0495 | 1.5449 | 0.6961 |  |  |
| Turicibacter | 0.8896 | 1.3571 | 0.8488 | 1.3525 | 1.5105 |  |  |
| Roseburia | 1.0788 | 0.6179 | 0.7763 | 0.8211 | 1.0789 |  |  |
| Catabacter | 0.4704 | 1.2233 | 0.5954 | 1.0233 | 0.4845 |  |  |
| Butyrivibrio | 0.6629 | 0.6829 | 0.7099 | 0.9439 | 0.6980 |  |  |
| Porphyromonas | 0.2873 | 0.7790 | 0.7116 | 0.8225 | 1.0124 |  |  |
| Anaerotruncus | 0.7370 | 0.4916 | 1.1113 | 0.8401 | 0.4223 |  |  |
| Faecalibacterium | 0.8802 | 0.6339 | 0.5309 | 0.7395 | 0.6579 |  |  |
| Alistipes | 0.5426 | 0.8382 | 0.7293 | 0.3967 | 0.5724 |  |  |
| Parabacteroides | 0.7562 | 0.3687 | 0.7249 | 0.3582 | 0.6235 | 0.1240 | 0.0986 |
| Phascolarctobacterium | 0.6442 | 0.6721 | 0.4064 | 0.6993 | 0.3955 |  |  |
| Succinivibrio | 0.8911 | 0.2260 | 0.3783 | 0.9182 | 0.2992 |  |  |
| Fibrobacter | 0.6833 | 0.3091 | 0.5231 | 0.5267 | 0.5104 |  |  |
| Hydrogenoanaerobacterium | 0.3134 | 0.6277 | 0.5472 | 0.7650 | 0.2653 | 0.1270 | 0.0679 |
| Subdoligranulum | 0.5588 | 0.4069 | 0.3513 | 0.5803 | 0.4173 |  |  |
| Pseudoflavonifractor | 0.3513 | 0.4729 | 0.3436 | 0.7103 | 0.1748 | 0.1220 | 0.0760 |
| Anaerovibrio | 0.3130 | 0.2380 | 0.2285 | 0.3668 | 0.6307 |  |  |
| Acetivibrio | 0.2050 | 0.4292 | 0.2038 | 0.3453 | 0.1883 | 0.0460 | 0.0961 |
| Paraprevotella | 0.1146 | 0.2449 | 0.4560 | 0.2155 | 0.2414 |  |  |
| Megasphaera | 0.2580 | 0.3105 | 0.2081 | 0.3017 | 0.1805 |  |  |
| Ethanoligenens | 0.1435 | 0.2909 | 0.2292 | 0.4047 | 0.1463 | 0.0480 | *0.0329 |
| Blautia | 0.1470 | 0.2936 | 0.3047 | 0.2621 | 0.1941 |  |  |
| Selenomonas | 0.1408 | 0.2591 | 0.1690 | 0.3491 | 0.1167 | 0.0370 | 0.0638 |
| Pseudobutyrivibrio | 0.1945 | 0.2773 | 0.1103 | 0.2278 | 0.2242 |  |  |
| Papillibacter | 0.1195 | 0.2014 | 0.2328 | 0.1745 | 0.1668 |  |  |
| TM7 (genus) | 0.0463 | 0.0507 | 0.2464 | 0.3909 | 0.0807 |  |  |
| Anaerovorax | 0.1087 | 0.1418 | 0.1665 | 0.3131 | 0.0662 |  |  |
| Ruminobacter | 0.6305 | 0.0000 | 0.0133 | 0.0000 | 0.1422 |  |  |
| Shigella | 0.4251 | 0.0298 | 0.0215 | 0.2420 | 0.0355 |  |  |
| Lachnobacterium | 0.1682 | 0.1846 | 0.1748 | 0.1220 | 0.1016 |  |  |
| Acetanaerobacterium | 0.0719 | 0.2504 | 0.1282 | 0.1340 | 0.1167 |  |  |
| Paludibacter | 0.1034 | 0.0322 | 0.2765 | 0.1186 | 0.1670 |  |  |
| Veillonella | 0.1199 | 0.1991 | 0.1124 | 0.1188 | 0.0855 |  |  |
| Desulfonispora | 0.0439 | 0.1155 | 0.1312 | 0.2250 | 0.0747 | 0.0190 | 0.0557 |
| Johnsonella | 0.1040 | 0.1631 | 0.0765 | 0.1781 | 0.0647 |  |  |
| Paenibacillus | 0.0579 | 0.1523 | 0.0722 | 0.1388 | 0.1150 |  |  |
| Sutterella | 0.1291 | 0.0211 | 0.0978 | 0.1793 | 0.0786 |  |  |
| Metabacterium | 0.0321 | 0.2034 | 0.0243 | 0.2184 | 0.0267 |  |  |
| Spirochaeta | 0.0270 | 0.0385 | 0.0715 | 0.0144 | 0.3192 |  |  |
| Lactobacillus | 0.0492 | 0.1003 | 0.0922 | 0.1303 | 0.0838 |  |  |
| Butyricimonas | 0.0323 | 0.0234 | 0.2987 | 0.0325 | 0.0672 |  |  |
| Butyricicoccus | 0.0472 | 0.0716 | 0.1284 | 0.1031 | 0.0821 |  |  |
| Acholeplasma | 0.0768 | 0.1143 | 0.1049 | 0.0495 | 0.0823 |  |  |
| Fusobacterium | 0.0739 | 0.0659 | 0.1003 | 0.1388 | 0.0455 |  |  |
| Barnesiella | 0.0773 | 0.0317 | 0.1230 | 0.0341 | 0.1217 | 0.0080 | 0.0793 |
| Alkalibaculum | 0.0709 | 0.1216 | 0.0309 | 0.1294 | 0.0342 |  |  |
| Percent of total abundance | 97.7287 | 96.9363 | 96.7684 | 96.6749 | 97.8558 |  |  |
