# Supplementary material for: Influence of wet distillers grains diets on beef cattle fecal bacterial community structure
Source: BMC Microbiol. 2012 Feb 24;12:25. doi: 10.1186/1471-2180-12-25 (PMC3305651; doi:10.1186/1471-2180-12-25)
Supplement: Additional file 13 — Table S4. Average abundance of species by treatment. Species that showed a response to dietary treatment (see SEM and P-values). [file 1471-2180-12-25-S13.DOC]

**Additional File 13,** Table S4 Average abundance of species by treatment. Species that showed a response to dietary treatment (see SEM and P-values).

| **Treatment by species** | **5S** | **10C** | **10S** | **15S** | **Con** | **SEM** | ***P*-value** |
| --- | --- | --- | --- | --- | --- | --- | --- |
| Clostridium sp | 7.6593 | 13.7312 | 9.8889 | 13.1241 | 8.8663 | 1.5850 | 0.0630 |
| Oscillospira sp | 7.7997 | 12.6798 | 10.1715 | 12.5771 | 6.8404 |  |  |
| Prevotella sp | 16.0338 | 5.0226 | 12.5640 | 5.5135 | 9.5759 |  |  |
| Bacteroides sp | 7.7122 | 8.8183 | 6.8411 | 6.7060 | 6.7497 |  |  |
| Ruminococcus sp | 2.9216 | 4.7132 | 4.0619 | 5.5543 | 3.4376 |  |  |
| Eubacterium sp | 2.9857 | 3.4287 | 5.1191 | 3.7003 | 3.2913 |  |  |
| Treponema sp | 2.9184 | 3.0624 | 0.9604 | 0.8525 | 9.6995 |  |  |
| Oscillibacter valericigenes | 3.5114 | 3.0964 | 2.6258 | 3.0618 | 2.3107 |  |  |
| Prevotella copri | 5.9991 | 0.8717 | 1.7124 | 0.4994 | 4.4178 |  |  |
| Tannerella sp | 1.7187 | 1.7409 | 4.5646 | 1.0569 | 2.5650 | 0.1220 | 0.0760 |
| Oscillibacter sp | 2.1133 | 2.8103 | 2.2149 | 2.5044 | 1.7042 |  |  |
| Coprococcus sp | 1.8226 | 2.3254 | 1.9429 | 1.6854 | 2.3138 |  |  |
| Bacillus sp | 1.1738 | 2.0622 | 1.2828 | 2.0485 | 1.2746 |  |  |
| Akkermansia muciniphila | 0.2788 | 0.5000 | 0.0475 | 0.1858 | 6.0548 |  |  |
| Anaerofilum sp | 1.3024 | 1.3922 | 1.2659 | 1.4613 | 1.2307 |  |  |
| Escherichia sp | 4.1327 | 0.1855 | 0.1309 | 1.3420 | 0.3485 |  |  |
| Ruminococcus flavefaciens | 0.4721 | 2.1430 | 1.5471 | 1.3752 | 0.5978 |  |  |
| Sporobacter sp | 1.1290 | 1.5913 | 1.0238 | 1.5323 | 0.6961 |  |  |
| Turicibacter sp | 0.8378 | 1.2629 | 0.8048 | 1.2603 | 1.4505 |  |  |
| Treponema porcinum | 0.1717 | 1.2188 | 1.1285 | 1.5601 | 1.4115 |  |  |
| Clostridium methylpentosum | 0.3846 | 1.0474 | 1.1283 | 1.1321 | 0.4449 |  |  |
| Oscillospira guilliermondii | 0.5250 | 0.6744 | 0.7259 | 1.0443 | 0.4885 |  |  |
| Porphyromonas sp | 0.2134 | 0.6798 | 0.5911 | 0.7513 | 0.8349 |  |  |
| Eubacterium siraeum | 0.3639 | 0.6576 | 0.5676 | 0.9446 | 0.3632 |  |  |
| Phascolarctobacterium sp | 0.6320 | 0.6195 | 0.4064 | 0.6803 | 0.3919 |  |  |
| Clostridium nexile | 0.4229 | 0.5545 | 0.6412 | 0.5481 | 0.5217 |  |  |
| Faecalibacterium sp | 0.6340 | 0.4905 | 0.4333 | 0.5704 | 0.5100 |  |  |
| Succinivibrio sp | 0.8490 | 0.2260 | 0.3783 | 0.8448 | 0.2952 |  |  |
| Anaerotruncus colihominis | 0.4638 | 0.2689 | 0.8305 | 0.5495 | 0.3279 |  |  |
| Eubacterium coprostanoligenes | 0.2566 | 0.5291 | 0.5966 | 0.6422 | 0.4056 |  |  |
| Ruminococcus albus | 0.2056 | 0.6156 | 0.6662 | 0.6138 | 0.3049 |  |  |
| Roseburia sp | 0.4652 | 0.3384 | 0.4523 | 0.4560 | 0.5493 |  |  |
| Butyrivibrio fibrisolvens | 0.3023 | 0.3961 | 0.4449 | 0.5474 | 0.3817 |  |  |
| Clostridium clariflavum | 0.2770 | 0.4867 | 0.3754 | 0.2545 | 0.6682 |  |  |
| Pseudoflavonifractor capillosus | 0.3513 | 0.4729 | 0.3436 | 0.7103 | 0.1748 | 0.1060 | *0.0089 |
| Clostridium orbiscindens | 0.2803 | 0.3850 | 0.3797 | 0.8002 | 0.1723 |  |  |
| Catabacter hongkongensis | 0.2428 | 0.7017 | 0.2939 | 0.4157 | 0.3049 |  |  |
| Prevotella stercorea | 0.9230 | 0.0190 | 0.4390 | 0.0868 | 0.4476 |  |  |
| Fibrobacter intestinalis | 0.6788 | 0.1695 | 0.3455 | 0.2435 | 0.4388 |  |  |
| Catabacter sp | 0.2276 | 0.5217 | 0.3014 | 0.6076 | 0.1796 | 0.1110 | 0.0623 |
| Hydrogenoanaerobacterium saccharovorans | 0.2418 | 0.4386 | 0.3821 | 0.5704 | 0.1908 | 0.0950 | 0.0788 |
| Clostridium symbiosum | 0.3369 | 0.2594 | 0.3579 | 0.2962 | 0.4803 |  |  |
| Anaerovibrio sp | 0.2963 | 0.2256 | 0.2161 | 0.3558 | 0.6047 |  |  |
| Eubacterium oxidoreducens | 0.2810 | 0.4139 | 0.3943 | 0.4029 | 0.1942 |  |  |
| Clostridium viride | 0.2566 | 0.4492 | 0.2515 | 0.4278 | 0.2800 |  |  |
| Prevotella marshii | 1.2797 | 0.0761 | 0.0696 | 0.0774 | 0.0927 |  |  |
| Butyrivibrio crossotus | 0.3566 | 0.2835 | 0.2491 | 0.3801 | 0.3123 |  |  |
| Treponema zioleckii | 0.0648 | 0.0322 | 0.2570 | 0.0037 | 1.2224 |  |  |
| Prevotella shahii | 0.2124 | 0.1570 | 0.7970 | 0.1603 | 0.2518 |  |  |
| Clostridium leptum | 0.2050 | 0.2385 | 0.4562 | 0.4142 | 0.2402 |  |  |
| Ruminococcus bromii | 0.2689 | 0.3418 | 0.2555 | 0.4919 | 0.1616 | 0.0620 | *0.0232 |
| Alistipes sp | 0.2445 | 0.5171 | 0.1859 | 0.2061 | 0.1858 |  |  |
| Subdoligranulum sp | 0.3190 | 0.2357 | 0.1691 | 0.3305 | 0.2847 |  |  |
| Megasphaera sp | 0.2531 | 0.3105 | 0.2038 | 0.3017 | 0.1734 |  |  |
| Acetivibrio sp | 0.1951 | 0.3810 | 0.1870 | 0.2979 | 0.1807 |  |  |
| Prevotella ruminicola | 0.2726 | 0.0956 | 0.4531 | 0.1810 | 0.2154 |  |  |
| Anaerotruncus sp | 0.2732 | 0.2227 | 0.2808 | 0.2906 | 0.0944 |  |  |
| Clostridium polysaccharolyticum | 0.4175 | 0.1094 | 0.2159 | 0.1006 | 0.3077 |  |  |
| Bacteroides acidifaciens | 0.1663 | 0.2757 | 0.3219 | 0.1574 | 0.1923 |  |  |
| Parabacteroides merdae | 0.3321 | 0.2018 | 0.2297 | 0.0748 | 0.2572 | 0.0520 | *0.0393 |
| Bacteroides uniformis | 0.1866 | 0.2429 | 0.2764 | 0.2397 | 0.1254 |  |  |
| Escherichia coli | 0.7516 | 0.0138 | 0.0382 | 0.1966 | 0.0633 |  |  |
| Ethanoligenens sp | 0.1200 | 0.2302 | 0.2087 | 0.3703 | 0.1272 |  |  |
| Roseburia inulinivorans | 0.4227 | 0.1046 | 0.0940 | 0.2472 | 0.1490 |  |  |
| Alistipes finegoldii | 0.2480 | 0.1260 | 0.4368 | 0.0637 | 0.1316 |  |  |
| Subdoligranulum variabile | 0.2398 | 0.1713 | 0.1823 | 0.2498 | 0.1326 |  |  |
| Clostridium glycyrrhizinilyticum | 0.1590 | 0.1940 | 0.1725 | 0.1483 | 0.2280 |  |  |
| Papillibacter cinnamivorans | 0.1195 | 0.2014 | 0.2328 | 0.1745 | 0.1668 |  |  |
| Parabacteroides sp | 0.2521 | 0.1138 | 0.1819 | 0.1222 | 0.2174 |  |  |
| Prevotella baroniae | 0.4359 | 0.0000 | 0.1541 | 0.1059 | 0.1793 |  |  |
| Clostridium herbivorans | 0.1811 | 0.2010 | 0.1258 | 0.2191 | 0.1130 |  |  |
| TM7 uncultured | 0.0463 | 0.0507 | 0.2464 | 0.3909 | 0.0807 |  |  |
| Faecalibacterium prausnitzii | 0.2462 | 0.1434 | 0.0976 | 0.1690 | 0.1479 |  |  |
| Pseudobutyrivibrio ruminis | 0.1613 | 0.1845 | 0.0712 | 0.1742 | 0.2049 |  |  |
| Anaerovorax sp | 0.1087 | 0.1418 | 0.1665 | 0.3131 | 0.0626 |  |  |
| Ruminobacter amylophilus | 0.5997 | 0.0000 | 0.0133 | 0.0000 | 0.1268 |  |  |
| Clostridium cellulosi | 0.1978 | 0.0973 | 0.0708 | 0.1400 | 0.2227 |  |  |
| Bacteroides barnesiae | 0.0727 | 0.1879 | 0.1946 | 0.1585 | 0.1093 |  |  |
| Coprococcus eutactus | 0.1119 | 0.1701 | 0.1156 | 0.1944 | 0.1153 |  |  |
| Acetanaerobacterium elongatum | 0.0719 | 0.2504 | 0.1282 | 0.1340 | 0.1167 |  |  |
|  | 92.3993 | 90.6014 | 89.3813 | 89.3772 | 91.7853 |  |  |
